# Graphene-Derived Carbon Support Boosts Proton Exchange Membrane Fuel Cell Catalyst Stability

**DOI:** 10.1021/acscatal.2c01753

**Published:** 2022-07-21

**Authors:** Luka Pavko, Matija Gatalo, Matjaž Finšgar, Francisco Ruiz-Zepeda, Konrad Ehelebe, Pascal Kaiser, Moritz Geuß, Tina Đukić, Angelja Kjara Surca, Martin Šala, Marjan Bele, Serhiy Cherevko, Boštjan Genorio, Nejc Hodnik, Miran Gaberšček

**Affiliations:** † Department of Materials Chemistry, National Institute of Chemistry, Hajdrihova 19, Ljubljana 1000, Slovenia; ‡ Faculty of Chemistry and Chemical Technology, University of Ljubljana, Ljubljana 1000, Slovenia; § ReCatalyst d.o.o., Hajdrihova 19, Ljubljana 1000, Slovenia; ∥ Laboratory for Analytical Chemistry and Industrial Analysis, Faculty of Chemistry and Chemical Engineering, University of Maribor, Smetanova ulica 17, Maribor 2000, Slovenia; ⊥ Helmholtz-Institute Erlangen-Nürnberg for Renewable Energy (IEK-11), Forschungszentrum Jülich GmbH, Cauerstr. 1, Erlangen 91058, Germany; # Department of Chemical and Biological Engineering, Friedrich-Alexander University Erlangen-Nürnberg, Egerlandstr. 3, Erlangen 91058, Germany; ∇ Department of Analytical Chemistry, National Institute of Chemistry, Hajdrihova 19, Ljubljana 1000, Slovenia

**Keywords:** PEMFC, durability, carbon support, reduced graphene oxide, mass transport

## Abstract

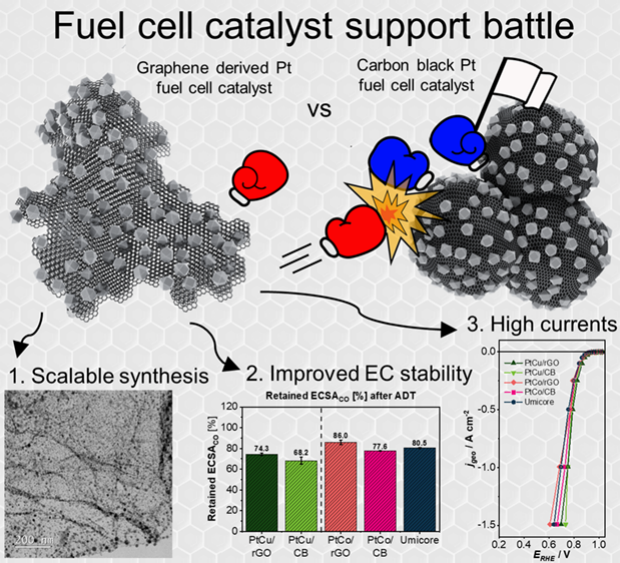

The lack of efficient and durable proton exchange membrane fuel cell electrocatalysts for the oxygen reduction reaction is still restraining the present hydrogen technology. Graphene-based carbon materials have emerged as a potential solution to replace the existing carbon black (CB) supports; however, their potential was never fully exploited as a commercial solution because of their more demanding properties. Here, a unique and industrially scalable synthesis of platinum-based electrocatalysts on graphene derivative (GD) supports is presented. With an innovative approach, highly homogeneous as well as high metal loaded platinum-alloy (up to 60 wt %) intermetallic catalysts on GDs are achieved. Accelerated degradation tests show enhanced durability when compared to the CB-supported analogues including the commercial benchmark. Additionally, in combination with X-ray photoelectron spectroscopy Auger characterization and Raman spectroscopy, a clear connection between the *sp*^2^ content and structural defects in carbon materials with the catalyst durability is observed. Advanced gas diffusion electrode results show that the GD-supported catalysts exhibit excellent mass activities and possess the properties necessary to reach high currents if utilized correctly. We show record-high peak power densities in comparison to the prior best literature on platinum-based GD-supported materials which is promising information for future application.

## Introduction

Proton exchange membrane fuel cells (PEMFCs) provide the zero-emission energy conversion solution critical to reaching complete decarbonization of the automotive and other energy sectors.^1^ However, widespread adoption of this technology is still limited by green hydrogen production capacities and its accessibility^2^ as well as several other factors directly related to the PEMFC technology itself.^3,4^ To unlock the full potential of PEMFCs, the catalyst material should be optimized in terms of increased performance, robustness, decreased usage of expensive and scarce platinum (Pt),^4,5^ and perhaps most importantly, increased long-term durability.^6,7^ In terms of performance, it is recognized that one of the major performance bottlenecks in the technology is the cathode oxygen reduction reaction (ORR).^8^ Currently, the best commercially available electrocatalysts for the ORR are Pt nanoparticles (NPs) or Pt-alloy NPs.^4^ To maximize the utilization of Pt, the catalyst is typically loaded onto various microporous carbon black (CB) supports, which, in addition to the electrical wiring of NPs and water management, also improves the mass transport of gaseous species.^9^ However, providing sufficient durability of such catalyst nanocomposites remains a significant challenge.^10^ Especially for the application in heavy-duty vehicles (HDVs), significant system lifetime improvements must be achieved to make PEMFC technology a viable option in this transport sector.^6^ There are two basic groups of catalyst composite degradation mechanisms: (i) electrochemically induced (transient) dissolution of Pt, which is closely related to the dynamics of formation/reduction of the Pt-oxide, resulting in Ostwald ripening and/or formation of metallic Pt bands in the membrane and (ii) electrochemical and chemical carbon support corrosion, leading to the agglomeration and/or detachment of Pt NPs.^11,12^ Both processes are interconnected, and it is almost impossible to discuss one without mentioning the other. Closer to the operating conditions of PEMFCs at elevated temperatures (60–80 °C) in the potential window of 0.6–1.0 V_RHE_, Pt dissolution is the more dominant degradation process, with carbon corrosion becoming more significant above 1.0 V_RHE_, which reflects the fuel cell start-up and shut-down conditions.^13^ In particular, the temperature is of significant importance when testing the durability of novel carbon-based supports, as the rate of carbon corrosion follows the Arrhenius law and increases exponentially with temperature.^14^ Nevertheless, both mechanisms ultimately lead to the lowering of the electrochemically active surface area (ECSA) of Pt and thus, loss of catalyst performance over time.

Graphene derivatives (GDs) such as graphene, reduced graphene oxide (rGO), and graphene nanoribbons possess specific chemical and physical properties compared to CBs. These include a higher specific surface area, better electronic conductivity, higher carbon *sp*^2^ content, a higher 2D crystallinity, and fewer structural defects.^15,16^ These properties of GDs translate to better thermodynamic stability.^17,18^ In principle, if GDs get appropriately exploited as supports for ORR catalysts, these benefits should provide significant improvements in terms of long-term durability resulting from increased resistance against carbon corrosion. This is of crucial importance to meet the ambitious targets of 30,000 h system lifetimes for HDV fuel cell applications.^6^

However, despite many efforts in recent years to utilize GDs as advanced PEMFC catalyst supports, several major challenges prevented any significant breakthroughs. Namely, in comparison to CB-supported catalysts that are usually prepared with some of the existing industrial water-based synthesis methods (e.g., the incipient wetness impregnation with chemical or thermal decomposition),^19^ the more hydrophobic nature of GD materials and their restacking tendency^20^ make it extremely difficult to achieve both a high metal loading (e.g., >30 wt %) and uniform distribution of Pt-based NPs and thus a sufficiently high ECSA. Both properties are necessary for appropriately high roughness factors of the catalyst layer and, subsequently, sufficient high current density performance.^7^ Furthermore, if one would use the oxidized version of the GD for NP deposition, improve hydrophilicity, and avoid restacking, the electronic conductivity of the carbon support would not meet the requirements for application in PEMFCs. Additional chemical reduction or thermal treatment of the carbon support in further steps to improve the electronic conductivity would cause additional structural defects to the GD support due to the loss of oxygen functional groups.^21^ This would lead to agglomeration and detachment of NPs, namely, loss in the ECSA.^7^ Consequently, new synthesis pathways are required to enable the synthesis of GD-supported analogues comparable with today’s state-of-the-art CB-supported Pt-based catalysts. This has led to many the strategies attempting to improve poor performance of the GDs using (i) various additives such as urea in the catalyst ink for the electrode preparation^22^ or (ii) using additives such as CBs to act as spacers that prevent restacking of GD-supported catalysts^23^ or (iii) synthesis of hybrid CB/GD^15,24−26^ or even MO_*x*_/GD-supported catalysts.^27^ Such strategies hypothesize the issues in the porosity of the catalyst layer and/or insufficient conductivity of the (usually) partly oxidized GD supports. Ideally, these issues should be resolved at the catalyst level alone.^7^

In the present study, we demonstrate a viable and scalable pathway to produce multigram quantities of high-performance GD-supported Pt-based ORR catalysts. Using this method, we are able to show that properly utilized GDs result in superior catalyst support, exhibiting both enhanced durability when compared to CB-supported analogues and the ability to reach a high current density performance comparable to that of CB-supported catalysts, which has not been the case until now. Furthermore, this study provides a fundamental explanation for the enhanced properties of GD-supported ORR catalysts.

## Results and Discussion

**Figure 1:**
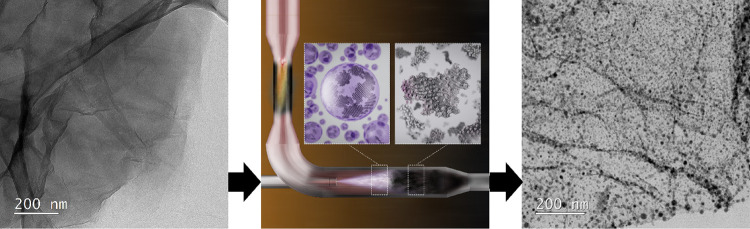
(a) Transmission electron microscopy (TEM) image of the GO, (b) scheme of the PC reactor where the left inset scheme is showing the M + GO suspension right before the formation of M/rGO (right inset scheme) and a narrow reaction zone between the insets, and (c) scanning transmission electron microscopy (STEM) image of the M/rGO showing high metal loading and uniform distribution of NPs.

In this study, the production of the GD-supported catalysts is based on further development of procedures published in two recent publications: (i) pulse combustion (PC) reactor technology^28^ for the production of a wide variety of multigram quantities of M/GD composites (M = Cu or Co; GD = rGO or reduced graphene oxide nanoribbons (rGONRs)) and then using (ii) double passivation with galvanic displacement (DP) methodology,^29^ enabling highly uniform deposition of Pt-based NPs on the GD supports. In the first step, graphite is used as a starting material for the synthesis of graphene oxide (GO) (Figure 1a) using the modified Hummer’s method described elsewhere^30^ (multiwalled carbon nanotubes have also been used for the preparation of GONRs see the Supporting Information for details). Because the oxidized GD materials exhibit hydrophilicity, this enables good interaction with M-salts such as the Co or Cu acetates used in the present study to create homogeneous aqueous M + GO/GONR suspensions.

The M + GO/GONR suspensions are then continuously fed through the PC reactor (Figure 1b). The PC synthesis step is continuous, with an extremely short reaction time (∼2 s), which in combination with the pulsating effect allows for every carbon primary particle to have well-defined and controlled reaction conditions. The PC synthesis results in (1) solvent evaporation, (2) partial thermal decomposition/reduction of GO/GONR toward rGO/rGONR, and (3) thermally induced decomposition of the M-salt that results in a very uniform distribution of very small M NPs over a carbon support (Figure 1c). The continuous operating mode of the PC reactor combined with a very short reaction time provides a key advantage over batch operating synthesis methods (see Supporting Information; Figure S1 for comparison). We note that the benefit of the fast partial thermal decomposition of oxidized GD in the (2) PC synthesis step is immediately followed by (3) thermally induced decomposition of the M-salt, which prevents the GDs from restacking and allows for the formation of a highly exfoliated, homogeneous M/GD material. Furthermore, even if a short reaction time could be reproduced in a batch mode setup, the reaction conditions would still not be as ideal as in continuous operating mode due to the varying local reaction conditions in the reaction vessel as the reaction proceeds. This would lead to the formation of a nonhomogeneous material, which would not meet the high requirements of a PEMFC electrocatalyst^7^ in further synthesis steps.

**Figure 2:**
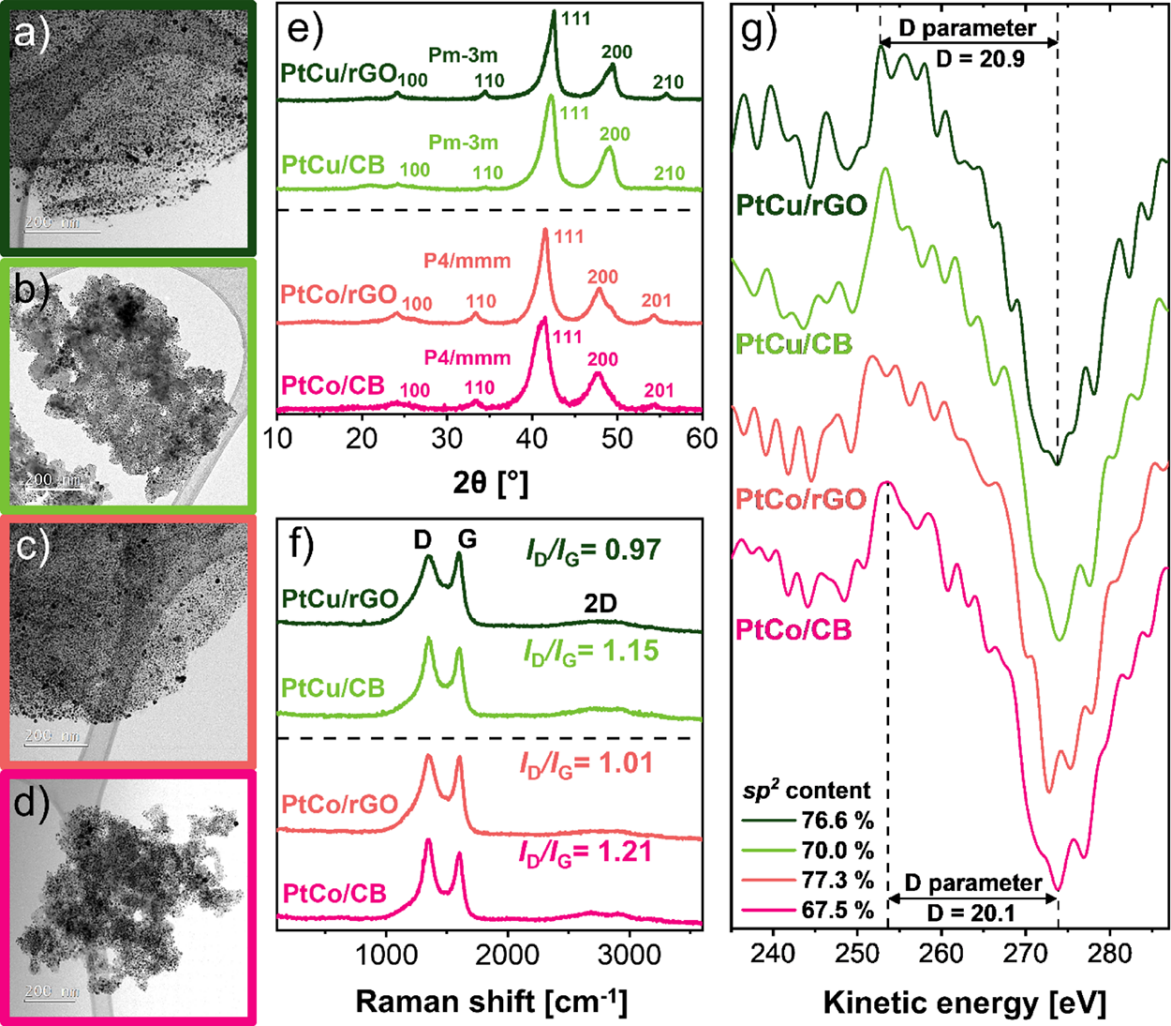
TEM images of the final intermetallic (a, c) PtM/GD and (b, d) PtM/CB electrocatalysts, (e) X-ray diffraction (XRD) patterns, (f) representative Raman spectra, and (g) first differential of the C (KLL) Auger spectra indicating the X-ray photoelectron spectroscopy (XPS)-derived D-parameter and the legend showing the calculated *sp*^2^ content values in %.

The PC approach, in combination with the Pt deposition using the DP methodology, provides an easily scalable commercial solution for the utilization of GDs as an advanced catalyst support. Thermal annealing^31^ is then used to alloy the remaining M to obtain the final intermetallic PtM ORR catalysts on both the CB and GD supports presented in Figure 2.

Figure 2 presents the structural comparison between the final PtM/GD catalysts (M = Cu or Co) supported on the reduced graphene oxide (rGO) and their CB analogues supported on Ketjen Black EC300J. To gain further understanding of the differences in the morphology of the various supporting materials prepared within this study, TEM images are presented in Figure 2a–d, for additional annular dark field (ADF) STEM characterization see Figures S2–5 as well as Figures S6–10 for scanning electron microscope (SEM) characterization. Microscopy not only reveals the very high uniformity but also the high density of the PtM NPs for all the analogues prepared within this study—see particle size distribution derived from TEM images (Figure S11). Most importantly, however, TEM microscopy reveals a clear structural distinction between rGO and CB-supported analogues. In the case of CB analogues, the structure consists of 3D primary carbon particle agglomerates with a diameter between 30 and 50 nm (Figures S7 and S10 for SEM images showing CB morphology). On the other hand, in the case of the final GD-supported catalysts, the carbon primary particles consist of 2D carbon structures in the case of rGO-supported analogues (Figures S2, S4, S6, S9) and a ribbon-like structure, evenly decorated with PtM NPs in the case of the rGONR-supported analogue (Figure S8).

In addition, the observed uniform particle size distributions (Figure S11) are in accordance with the wide XRD peaks corresponding to the PtM crystal phases (Figures 2e and S12). The comparison of the XRD patterns shows that PtCu analogues exhibit the presence of the cubic (Pm-3 m) PtM_3_ intermetallic phase, whereas PtCo analogues exhibit the tetragonal PtM intermetallic phase (P4-mmm). In addition, XRD patterns of all the GD analogues prepared within this study are shown in Figure S12. It can be observed that regardless of the type of carbon support used, the diffraction peaks corresponding to the metallic phases of these analogues are located at almost identical *2*θ positions and exhibit a near-identical width of the most intense peaks. Hence, while on the one hand the combination of PC^28^ and DP^29^ methods allows one to vary the carbon support (e.g., various CBs or GDs); on the other hand, this synthesis approach is still flexible enough to enable precise control over other parameters such as the metal loading and Pt:M chemical composition (for exact metal loadings refer Tables S1–2). XRD provides for the “fingerprints” of the crystal phases related to the PtM NPs, whereas Raman spectra (Figure 2f) can serve as “fingerprints” for catalyst’s carbon supporting materials. A closer inspection of Raman spectra in Figure 2f of the D and G bands reveals a major difference between the CB and GD-supported analogues. Namely, an altered *I*_D_/*I*_G_ ratio, which is proportional to the level of defects present in the material, is visible between GD and CB-supported catalysts. In particular, for both presented GD-supported catalysts the *I*_D_/*I*_G_ ratio is approximately 1, whereas the *I*_D_/*I*_G_ ratio for CB-supported analogues is noticeably higher, that is, 1.15 and 1.21 for PtCu/CB and PtCo/CB analogues, respectively (see Figures S13 and S14 for the additional Raman spectra as well as spectra deconvolution). This is in accordance with the prior studies, as GDs are expected to contain fewer structural defects and a higher degree of graphitization compared to partially graphitized CBs.^32^Figure 2g shows the first derivative of the Auger C KLL spectra with the determined D-parameter for *sp*^2^ carbon content determination in the catalyst support. The *sp*^2^ content was calculated based on the linear relationship between the extremes of pure *sp*^2^ and *sp*^3^ C atoms by employing the eV difference between the minimum and maximum on the first derivative of the Auger C KLL spectra.^33,34^ The value of the *D* parameter is noticeably higher for both rGO-supported catalysts compared to the CB-supported analogues. It is evident that the rGO-supported catalysts also have a higher *sp*^2^ content (Figure 2g): 76.6% for PtCu/rGO and 77.3% for PtCo/rGO compared to only 70.0 and 67.5% for PtCu/CB and PtCo/CB analogues, respectively (XPS data given in the Supporting Information: Figures S15–17). This strongly agrees with the previous report^21^ and corresponds well with the Raman spectra (Figure 2f) where the rGO-supported catalysts possess fewer *sp*^3^ structural defects in carbon.^35^ These differences in the properties of the carbon support could translate into differences in long-term durability and performance of the catalysts, as demonstrated and discussed in continuation.

To adequately assess the durability of the synthesized GD-supported catalysts, an in-house designed high-temperature disc electrode (HT-DE) setup was used (Figure S18).^14,36^ The HT-DE setup enables performing accelerated degradation tests (ADTs) at an elevated temperature by using a reflux cooling condenser to avoid evaporation of the electrolyte (in this case 0.1 M HClO_4_). The use of high temperature provides the crucial parameter resulting in a severe stress test that exposes weaknesses of intrinsically less stable catalysts. Furthermore, ECSA values normalized via CO-electrooxidation (ECSA_CO_) and mass activity (MA) before and after the ADT are evaluated using a typical thin-film rotating disc electrode setup (TF-RDE). For the present study, rather harsh ADT conditions were used in the HT-DE setup that consisted of 5000 cycles in a potential window of 0.4–1.2 V_RHE_ with a scan rate of 1 V s^–1^. Most importantly, the experiments were run at an elevated temperature of 60 °C to increase the rate of carbon corrosion and also to simulate the real operating temperature of a PEMFC.^1^ For the present study, the most important parameter used for the assessment of durability was “the ability of the catalyst to retain the ECSA_CO_”. Recently, the crucial importance of both the temperature and the potential window for evaluation of the stability of the Pt-based carbon-supported catalysts has been clearly demonstrated.^36^ In this study, however, the initial focus went into the synthesis and preparation of catalyst analogues that are in many aspects as similar as possible with the only difference being the type of catalyst’ carbon-based support. In other words, using the same ADT parameters for all the analogues, the contribution related to the dissolution of metals should, for the most part, be very similar and thus, the main observed difference in retained ECSA_CO_ should be related to the differences in the carbon support. With that in mind, our main hypothesis before the durability investigation has been that GD-supported catalysts should exhibit a better ability to retain ECSA_CO_ with respect to the CB-supported analogues due to the increased content of *sp*^2^ carbon and fewer carbon structural defects.

**Figure 3:**
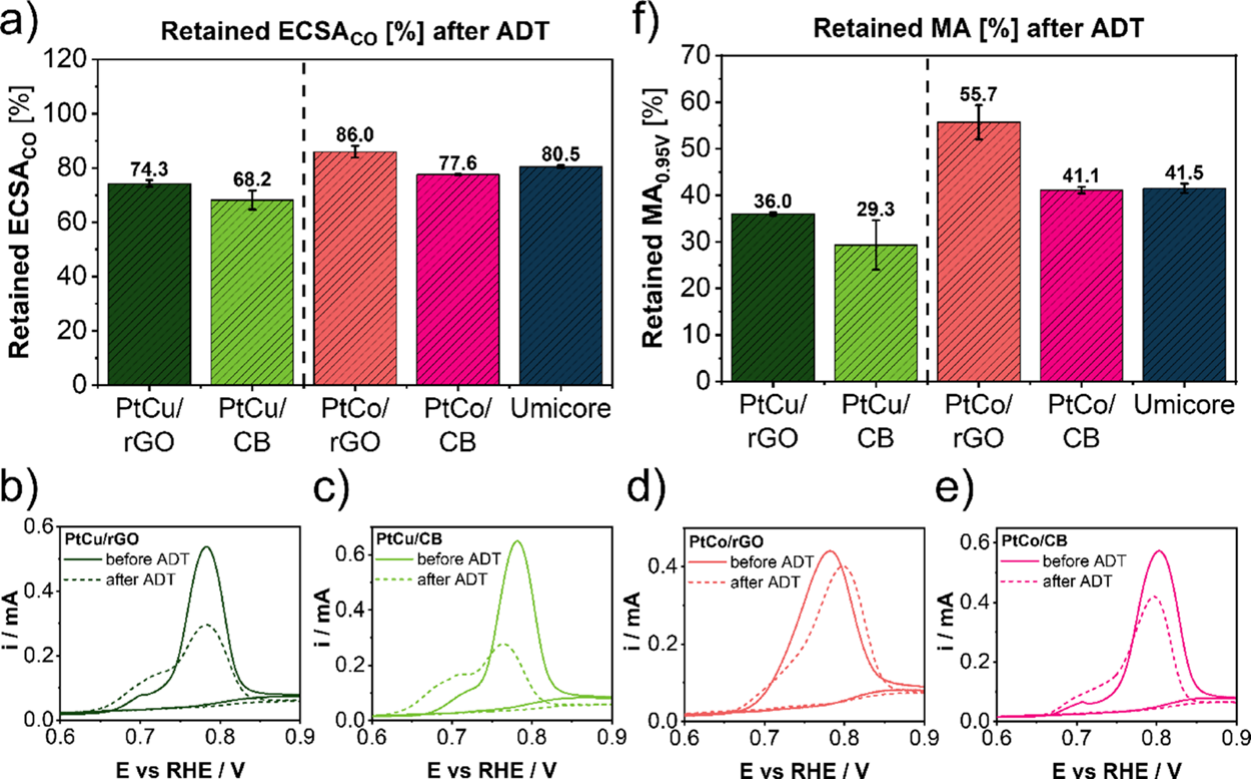
(a) Retained ECSA_CO_ after the ADT (5000 cycles at 60 °C between 0.4 and 1.2 V_RHE_, 1 V s^–1^ in 0.1 M HClO_4_), (b–e) CO-electrooxidation peak comparison before and after the ADT for the PtCu group (b, c) and PtCo group of catalysts (d–f) Retained MA after the same ADT conditions.

Figure 3a presents the ECSA_CO_ retention of the catalysts after the ADT (ECSA_CO_ values before and after the ADT are presented in Figure S19). For additional comparison and validation of durability, also a state-of-the-art commercial PtCo/CB benchmark from Umicore (Elyst Pt30 0690) denoted in Figure 3 as “Umicore” was also tested for durability. First, it can be observed that rGO-supported catalysts retained a higher ECSA_CO_ after the performed ADT when compared to their respective CB analogues (CO-electrooxidation peak comparison in Figure 3b–e, for full CO-stripping graphs, see Figure S20). Moreover, the PtCo/rGO samples with an ECSA_CO_ retention of 86.0% exhibited the highest durability among all the tested samples, including the commercial benchmark. Additionally, it can be observed that PtCu analogues retained less ECSA_CO_ than the PtCo analogues. The differences between both groups of Pt-alloys could be attributed to the better stability of the more Pt-rich PtM crystal structure in the case of PtCo analogues with respect to the PtM_3_ structure of the PtCu analogues (see Table S1).^37^ On the other hand, the intrinsic differences between PtCu and PtCo alloys or the intermetallic phase itself (cubic vs tetragonal) could play an important role as well.^38−40^ The durability comparison should thus only be made within the PtCu group or PtCo group of analogues, respectively. The origin of better catalyst durability should arise from differences between GD and CB carbon supports. Namely, the contents of structural defects measured with Raman spectroscopy (Figure 2f) and *sp*^2^ carbon derived from XPS C (KLL) Auger spectra (Figure 2g) differ drastically between the GD and CB. A higher *sp*^2^ carbon content and fewer structural defects, that is, a lower *I*_D_/*I*_G_ ratio, provide for improved carbon support stability in both PtCu and PtCo groups of materials. This indicates that, most likely, these two parameters play a crucial role also in the overall catalyst electrochemical durability after the ADT.

Figure 3f shows the MA retention in % determined at 0.95 V_RHE_ (MA values before and after the ADT are presented in Figure S21). It can be observed that similarly to ECSA_CO_ retention, the GD-supported catalysts also retained a higher MA after the ADT in comparison to CB-supported analogues. Moreover, the PtCo/rGO sample with a MA retention of 55.7% exhibited the highest value among all the tested samples, including the commercial benchmark which retained only 41.5% of the original MA. Additionally, it can also be observed that the MA retention values for the PtCo group of electrocatalysts are substantially higher than for the PtCu analogues. This is also in agreement with the XRD data that show a more dominant presence of a less stable ordered PtCu_3_ intermetallic phase in comparison to the more stable ordered PtCo intermetallic phase present in the PtCo line of materials.^38−40^ In general, for both groups of materials, it can be concluded that the ECSA_CO_ and the MA retention show on average higher values for GD-supported electrocatalysts when compared to the values of CB-supported analogues. The origin of the higher durability could, in principle, also be attributed to the different metal–support interaction (MSI) between the NPs and the carbon. A higher *sp*^2^ carbon content and fewer structural defects (lower *I*_D_/*I*_G_ ratio) of GD materials could provide for different MSI that favors better ECSA_CO_ as well as MA retention after the ADT. Although the exact correlation of MSI and its effect on the durability of the PEMFC catalysts is not yet well understood and defined, the results within this study suggest that this parameter could play an important role in understanding the origin of better catalyst durability and should therefore be further addressed in upcoming studies.^41,42^

**Figure 4:**
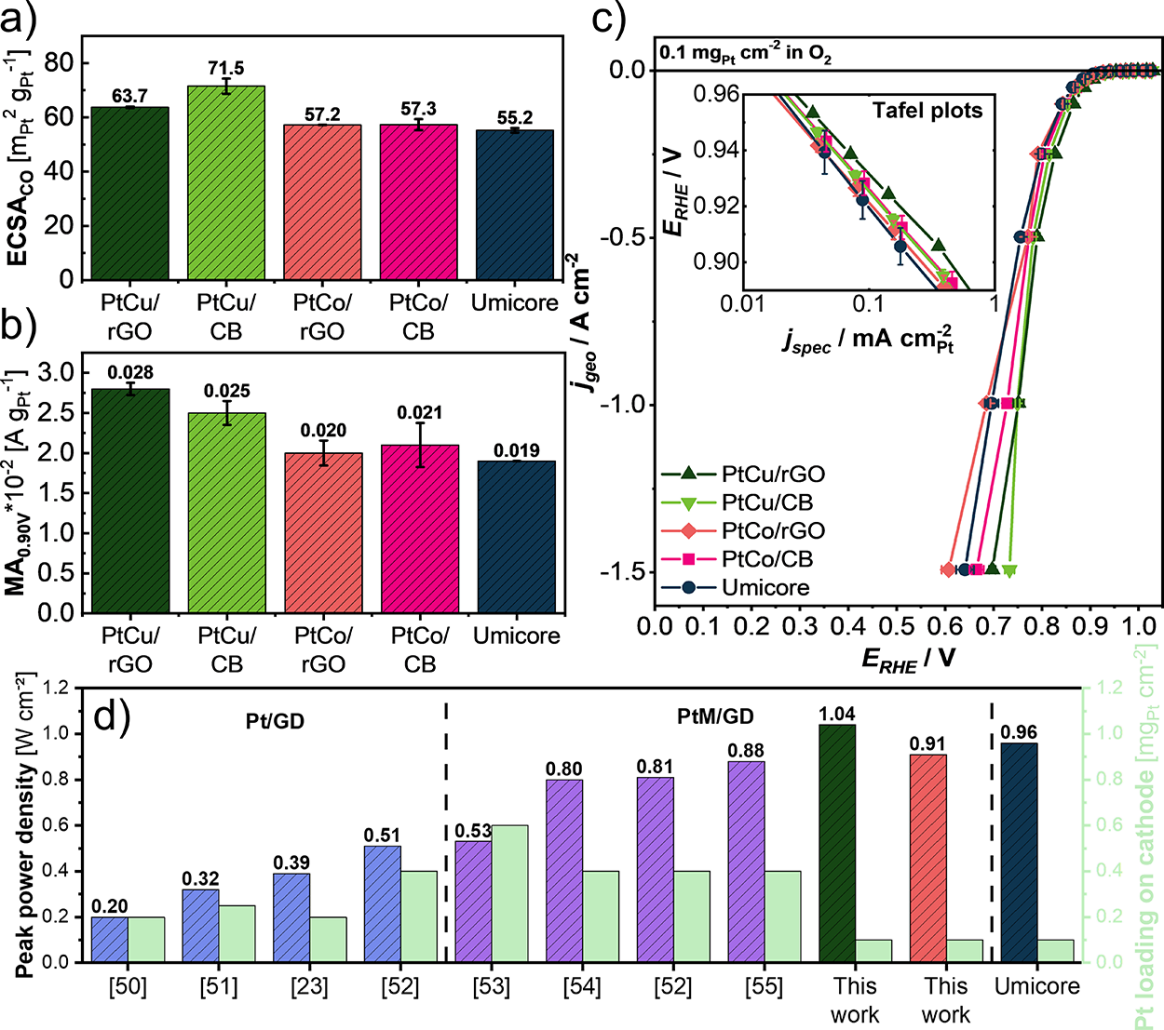
(a) ECSA values measured in the GDE half-cell setup, (b) values of mass activity at 0.90 V_RHE_ measured in the GDE half-cell setup, (c) ORR polarization curves showing high current density performance of the catalysts with inset figure showing Tafel plots, all measured in the GDE half-cell setup, and (d) comparison of the best peak power density performance of GD-supported catalysts found in the literature.^23,50−55^

The other historically difficult challenge is related to the performance of GD-supported Pt-based catalysts at PEM-FC relevant high current densities, especially for graphene-derived materials. To assess this in the present work, the gas-diffusion electrode (GDE) half-cell approach was used.^43,44^ This method has been proposed as a suitable tool to bridge the gap between the fundamental electrochemical catalyst evaluation using rotating disk electrode half-cell methods and the applied fuel cell research in single cells.^45−48^ Here, for additional validation of the catalysts’ performance, similarly to the previous chapter, a comparison with a commercial state-of-the-art PtCo/CB benchmark from Umicore (Elyst Pt30 0690) marked as “Umicore” in Figure 4 was also used. A very low Pt loading of only 0.1 mg_Pt_ cm^–2^ was used for all measurements, which is in accordance with Department of Energy (DoE) 2025 targets.^49^ Furthermore, the catalyst ink composition used in this study was based on the procedure optimized as part of the prior work.^43^ Namely, the ionomer-to-catalyst weight ratio was kept constant for all five catalysts evaluated in the GDE within this study. Thus, no further ink optimization was performed to obtain the results presented in continuation.

Figure 4a compares the obtained ECSA_CO_ values measured in the GDE half-cell. The values are comparable within the individual group of samples (PtCu and PtCo) and correlate well with the data obtained using the RDE (Figure S19a). It can also be noted that the values of ECSA_CO_ for samples prepared within this study exceed the values of the commercial benchmark (Elyst Pt30 0690). Additionally, the ECSA_CO_ values for PtCu samples are higher than those for the PtCo samples, which is also in strong agreement with the fact that PtCu samples possess different intermetallic phases (PtM_3_) in comparison to PtCo analogues (PtM) resulting in a higher ECSA_CO_ due to a lower Pt content in the core of NPs. Figure 4b, on the other hand, compares the MA of all the measured catalysts at 0.90 V_RHE_. It can be observed that the GD analogues possess a higher MA than the commercial benchmark (Elyst Pt30 0690). Similarly, as with ECSA_CO_ data, the higher values in MA for PtCu catalysts can be attributed to different intermetallic phases (PtM_3_ vs PtM) due to the more pronounced crystal lattice strain effect.^56^ Furthermore, by looking at the Tafel plots (inset in Figure 4c), it can be observed that the PtCu/rGO catalyst exhibits the best performance in the whole kinetic region among all the catalysts. This could mean that in this case, the carbon support has a positive effect on the stability of the electrocatalyst presented in Figure 3 and its kinetic activity measured in the real GDE catalyst layer which could in theory also be attributed to the different MSI.^41,42^

The ORR polarization curves shown in Figure 4c reveal important differences in the performance of the catalysts. Below the current density of cca. 0.5 A cm^–2^, the differences are somewhat less pronounced, but still, it can be concluded that the PtCu/rGO catalyst shows the best and the commercial benchmark (Elyst Pt30 0690) the worst performance. With increasing current density, the differences in the performance also increase. At a current density of 1.0 A cm^–2^ still the PtCu/rGO remains the best performing catalyst, whereas PtCo/rGO exhibits the worst performance. At current densities of 1.5 A cm^–2^, the difference in the performance becomes even more pronounced. Both CB analogues appear to slightly outperform their respective GD analogues. Nevertheless, at this point, it should be stressed once again that no catalyst ink optimization has been performed for GD-supported catalysts. This means that there could be room for improvement in high current density performance by optimizing the catalyst ink and consequently the catalyst layer alone. Hence, as a starting point, it seems that PtM/GD catalysts with a sufficiently high ECSA and the metal loading can reach high currents if appropriately utilized. The values of peak power density performance of GD-supported materials derived from already published work are collected in Figure 4d. It can be observed that the value of the GD supported catalyst prepared in this work is the highest among all, including the commercial CB-supported reference Umicore (Elyst Pt30 0690). When comparing the results from this work with the values from the literature, one needs to note the differences in Pt loading on the cathode. Here, a much smaller loading of only 0.1 mg_Pt_ cm^–2^ was used which is already meeting the automotive DoE 2025 targets^49^ and is on average 3–4 times smaller than used in the other publications. For instance, the best prior studies in the literature^52,55^ showcased peak power density performances of 0.81 and 0.88 W cm^–2^ for their GD-supported materials, respectively, which is still lower than the value of 1.04 W cm^–2^ reached in this work. However, whereas those previous studies reported Pt loadings of 0.4 mg_Pt_ cm^–2^, we used a very low loading of 0.1 mg_Pt_ cm^–2^ in the present work. Thus, the compared normalized values are expected to be even much more in favor of the present work. Namely, it is known that with increasing Pt loading on the cathode, the differences in the increased catalyst layer thickness start to affect the performance, increasing the mass transport resistance, which could hinder the catalyst’s performance.^57^ However, it has to be mentioned that here MEA single-cell results from the literature are compared to data obtained in a GDE half-cell. Hence, several parameters such as operating temperature, back pressure, and humidification can vary. Additionally, the effect of the hydrogen oxidation reaction at the anode (which can be considered minor in the PEMFC) is not included in GDE half-cell measurements. Nevertheless, a crucial barrier in the performance of the GD-supported materials in realistic catalyst layers has been overcome within this work.

## Conclusions

In conclusion, the applicability of GDs as an advanced support for Pt-based fuel cell electrocatalysts has been critically evaluated. Two groups of electrocatalysts, one based on PtCu and the other on the PtCo intermetallic catalyst, were prepared using the PC synthesis in combination with the double passivation with galvanic displacement method. This innovative and scalable synthesis approach allowed the preparation of very high loadings (up to 60 wt %) of uniformly dispersed metal NPs on GDs. Accelerated degradation testing using a high-temperature liquid electrolyte disc electrode showed that the rGO-supported catalysts exhibited enhanced electrochemical stability in both the ECSA and the mass activity retention compared to their CB-supported analogues, including the commercial benchmark (Elyst Pt30 0690). The XPS and Raman results indicate that the improved electrocatalyst durability is related to the increased content of *sp*^2^ carbon and the decrease of structural defects present in the rGO carbon support. Moreover, these differences could lead to an altered MSI, which could affect improved durability as well as the performance of the GD-supported catalysts. To evaluate the high current density performance, activity was measured in a GDE half-cell. On average, both electrocatalysts based on the rGO exhibited excellent kinetic performance as well as high current density performance, relevant for industrial application. Moreover, the peak power density values of the rGO-supported materials prepared in this work overpass the values from prior publications and the state-of-the-art commercial Pt-Co benchmark from Umicore (Elyst Pt30 0690). This makes rGO-based materials if utilized appropriately, a very promising candidate for a potential industrial application as carbon catalyst supports and should spark further investigation in this direction. In addition to real fuel cell tests and tuning of the catalyst layer’s mass transport, future optimizations of GD-derived materials could be achieved based on a deeper fundamental understanding of the origins of superior kinetic activity and stability.

## Supporting Information Available

Experimental procedures, SEM images, ADF and BF STEM images, particle size distributions, additional XRD patterns, EDX analysis, ICP-OES analysis, additional Raman characterization, XPS characterization, electrochemical measurements which include CO-electrooxidation CVs, and additional discussion (PDF)
