# Supplementary material for: Graphene-Derived Carbon Support Boosts Proton Exchange Membrane Fuel Cell Catalyst Stability
Source: ACS Catal. 2022 Jul 21;12(15):9540–8. doi: 10.1021/acscatal.2c01753 (PMC9361283; doi:10.1021/acscatal.2c01753)
Supplement: Supplementary file 1 — cs2c01753_si_001.pdf [file cs2c01753_si_001.pdf]

Supporting information

Graphene-Derived Carbon Support Boosts Proton Exchange Membrane Fuel Cell Catalyst Stability

Luka Pavko[a,b], *Matija Gatalo*[a,c,*], *Matjaž Finšgar*[d], *Francisco Ruiz-Zepeda*[a], *Konrad Ehelebe*[e,f], *Pascal Kaiser*[e,f], *Moritz Geuß*[e,f], *Tina Đukić*[a,b], *Angelja Kjara Surca*[a], *Martin Šala*[g], *Marjan Bele*[a], *Serhiy Cherevko*[e], *Boštjan Genorio*[b,*], *Nejc Hodnik*[a], *Miran Gaberšček*[a,*]

[a] Department of Materials Chemistry, National Institute of Chemistry, Hajdrihova 19, 1000 Ljubljana, Slovenia

[b] Faculty of Chemistry and Chemical Technology, University of Ljubljana, 1000 Ljubljana, Slovenia

[c] ReCatalyst d.o.o., Hajdrihova 19, 1000 Ljubljana, Slovenia

[d] Laboratory for Analytical Chemistry and Industrial Analysis, Faculty of Chemistry and Chemical Engineering, University of Maribor, Smetanova ulica 17, 2000 Maribor, Slovenia

[e] Helmholtz-Institute Erlangen-Nürnberg for Renewable Energy (IEK-11), Forschungszentrum Jülich GmbH, Cauerstr. 1, 91058 Erlangen, Germany

[f] Department of Chemical and Biological Engineering, Friedrich-Alexander University Erlangen-Nürnberg, Egerlandstr. 3, 91058 Erlangen, Germany

[g] Department of Analytical Chemistry, National Institute of Chemistry, Hajdrihova 19, 1000 Ljubljana, Slovenia

Corresponding authors.

Email: matija.gatalo@ki.si, bostjan.genorio@fkkt.uni-lj.si, miran.gaberscek@ki.si.

Experimental section

Graphene oxide, graphene oxide nanoribbons synthesis

Graphene oxide (GO) and graphene oxide nanoribbons (GONR) synthesis was based on a modified Hummer's method described elsewhere.[1] For this specific study, 1000 mL of sulfuric acid 96 wt% (Carlo Erba) was added to a 5 L beaker for all materials. Slowly 110 mL of phosphoric acid 85 wt% (Carlo Erba) was added. PTFE anchor stirrer was centered to the middle and set to 150 rpm using IKA RW16 basic overhead stirrer. 20 g of graphite KS44 series (Imerys) or M-grade multiwall carbon nanotubes (NTL) were slowly added to the mixture, followed by slow addition of 1 wt. eq. (~ 20 g) of $KMnO_4$ (Acros Organics) after 1 hour of stirring. This has been repeated 4 more times every 24 hours, adding up to a total 5 wt. eq. of $KMnO_4$. Afterward, the mixture was left to stir for an additional two days. In the next step, under continuously stirring, the reaction mixture was quenched *via* direct addition of 3 L of ice, followed by slow addition of 30 wt% H_2O_2 (Belinka) until the color changed from purple to yellowish. The stirring was then stopped and GO/GONR was then left to settle on the bottom of the reaction mixture. This was followed by discarding of the supernatant that was replaced with fresh ultrapure water (resistivity 18.2 MΩ cm, obtained from Milli-Q Direct Water Purification System, MilliPore). Lastly, GO/GONR was subjected to several washing steps. In the first step, GO/GONR suspension was centrifuged for 30 minutes at 10500 rpm (Sorvall LYNX 4000, Thermo Scientific) to achieve sedimentation of GO/GONR and discard the supernatant. In the second step, GO/GONR was re-dispersed in 5 wt% HCl prepared from 37 wt% HCl stock solution (Carlo Erba) for three h to dissolve any residual metals. The mixture was centrifuged at 10500 rpm for 30 minutes to remove the supernatant. The last cleaning step comprised re-dispersing GO/GONR in ultrapure water and soaking until the next day, followed by centrifugation at 10500 rpm for 1 h to discard the supernatant. This has been repeated 4 more times, adding up to a total of 5 washing cycles with ultrapure water. After the final supernatant was discarded, GO/GONR was again re-dispersed in ultrapure water with an approximate concentration of ~14 g_{GO} L^{-1} and treated with a homogenizer (Ultra-turrax T-25 basic, IKA) for 1 h at max rpm setting. GONR suspension was treated only with an ultrasonic bath (Iskra Sonis 4, Iskra) for 15 min.

Pulse combustion reactor synthesis

The exact procedure is already published elsewhere.[2] In the case of the synthesis of carbon black supported M/C composite materials, the feedstock suspension consisted of suspending 30 g carbon black (Ketjen Black EC300J) and a metal acetate hydrate in 1500 mL ultrapure water (resistivity 18.2 MΩ cm, obtained from Milli-Q Direct Water Purification System, MilliPore). In the case of Co/C composite, 75.4 g of cobalt acetate tetrahydrate (Sigma Aldrich) was used and in case of Cu/C composite, 60.5 g of copper acetate monohydrate (Sigma Aldrich). For the synthesis of Co/rGO composite, the feedstock suspension consisted of dissolving 25.0 g of cobalt acetate monohydrate (Sigma Aldrich) in 1 L of ~14 g_{GO} L^{-1} ultrapure water suspension, and in the case of Cu/rGO composite, the feedstock suspension consisted of dissolving 21.0 g of copper acetate monohydrate (Sigma Aldrich) in 1 L of ~14 g_{GO} L^{-1} ultrapure water suspension. Lastly, all feedstock suspensions were mixed vigorously using a homogenizer (Ultra-turrax T-25 basic, IKA) for 10 minutes before being introduced to the PC reactor. Each feedstock suspension was then continuously stirred with a mechanic stirrer (IKA) while being fed into the reactor using a peristaltic pump. Specific details regarding the working principle of pulse combustion reactor have already been published elsewhere.[3–5] During the synthesis, supported metal nanoparticles (SMNPs) are collected with the electrostatic filter, mounted behind the reaction pipe.

Synthesis of intermetallic Pt-alloy electrocatalysts

Both the CB and GD supported intermetallic Pt-alloy analogues were prepared in accordance with the processes already reported previously.[6] Briefly, the electrocatalysts have been prepared in two steps. In the first step, Pt NPs were deposited onto the M/C or M/GD composites by using a part of the base metal M as sacrificial *via* the double passivation galvanic displacement method reported.[6] In the second step, the prepared composites with deposited Pt NPs were thermally annealed in order to obtain an intermetallic crystal phase.[7] For the purpose of GDE evaluation, all the evaluated catalysts were also acid washed (de-alloyed) in accordance to the work described previously.[8,9]

ICP-OES and digestion

All reagents used were of analytical grade or better. For sample dilution and preparation of standards, ultrapure water (resistivity 18.2 MΩ cm, obtained from Milli-Q Direct Water Purification System, MilliPore) and ultrapure acids (HNO_3 and HCl, Merck-Suprapur) were used.

Standards were prepared in-house by dilution of certified, traceable, inductively coupled plasma (ICP)-grade single-element standards (Merck CertiPUR). A Varian 715-ES ICP optical emission spectrometer (OES) was used. Before ICP-OES analysis, each electrocatalyst was weighted (approximately 10 mg) and digested using a microwave-assisted digestion system (Milestone, Ethos 1) in a solution of 6 mL HCl and 2 mL HNO_3. Samples were then filtered, and the filter paper was again submitted to the same digestion protocol. These two times digested samples were cooled to room temperature and then diluted with 2 V% HNO_3 until the concentration as required.

XRD analysis

The powder X-ray diffraction (XRD) measurements of samples containing Ni and Cu were carried out on a PANalytical X'Pert PRO MPD diffractometer with Cu Kα1 radiation (λ = 1.5406 Å) in the 2θ range from 10° to 60° with the 0.034° step per 100 s using full opened X'Celerator detector. Samples were prepared on zero-background Si holder.

The powder X-ray diffraction (XRD) measurements of samples containing Co were carried out on a PANalytical X'Pert PRO diffractometer with Cu Kα radiation (λ = 1.541874 Å) in the 2θ range from 10° to 60° with the 0.039° step per 300 s using full opened Pixel detector. Samples were prepared on zero-background Si holder.

Transmission Electron Microscopy (TEM) and scanning TEM (STEM) analysis

TEM and STEM imaging was carried out in a probe Cs-corrected scanning transmission electron microscope Jeol ARM 200 CF operated at 80 kV. Different regions of the samples were inspected in order to gather information from the most representative parts. For STEM analysis powder samples were transferred to lacey-carbon coated copper or nickel grids. TEM images were used to do the particle size distribution using ImageJ software.

Raman characterization

The Raman spectra were recorded in the spectral range from 50 to 3700 cm^{-1} using an Alpha 300 confocal Raman spectrometer (WITec, Ulm, Germany) with 20x or 50x objective. Green laser with an excitation wavelength of 532 nm was used with a laser power ranging from 0.1 to 1 mW. The spectra were recorded up to 100 scans and integration times from 1 to 20 s depending on the sample. For each sample, three different locations were analyzed to verify the spectra.

Scanning electron microscope (SEM) and energy dispersive X-ray (EDX) analysis

Scanning electron microscope (SEM) analysis was performed on SUPRA 35 VP (Carl Zeiss) microscope at 5 kV using In-lens detector. Standard SEM pin mounts (Agar scientific) covered with conductive carbon tape (Agar scientific) were used to hold the powder samples.

Energy dispersive X-ray (EDX) analysis was performed using detector SDD Ultim max 100 (Oxford, UK) at 5 kV for PtCu samples and 20 kV for PtCo samples. Samples were prepared using the following procedure: Small amount of powder electrocatalyst sample (1-3 mg) was put on 13 mm polished metal disk and covered with the metal disk of the same size. Samples were pelleted with the manual press until the pellet with a thickness of about 50 µm was obtained. Standard SEM pin mounts (Agar scientific) covered with conductive carbon tape (Agar scientific) were used to hold pelleted samples.

X-ray photoelectron spectroscopy (XPS) analysis

X-ray photoelectron spectroscopy (XPS) was performed with the AXIS supra+ instrument (Kratos, Manchester, UK) using a monochromatic Al K_α X-ray source. The powder samples were immobilized on conductive carbon tape (Agar scientific) attached to a Si wafer (Agar scientific). The powder samples completely covered the entire surface of the carbon tape. The samples prepared in this way were fixed on the sample holder with conductive carbon tape. For each sample, spectra were acquired on a 300 by 700 µm analysis spot size. Survey spectra were measured at pass energy of 160 eV and an emission current of 15 mA, while high-resolution (HR) spectra were measured at pass energy of 20 eV and an emission current of 15 mA. For the measurements of the HR Auger C KLL spectra, at least 15 sweeps were performed to improve the S/N ratio as these spectra are located at relatively high binging energy.

Electrochemical evaluation via Thin Film Rotating Disc Electrode (TF-RDE)

Preparation of thin films and the setup – Electrochemical measurements were conducted with a CompactStat (Ivium Technologies) in a two-compartment electrochemical cell in a 0.1 M $HClO_4$ (Merck, Suprapur, 70 wt%, diluted by ultrapure water (18.2 MΩ cm)) electrolyte with a conventional three-electrode system (**Figure S16a**). Hydrogen electrode (Gaskatel) was used as a reference and a graphite rod as a counter electrode. The working electrode was a glassy carbon disc embedded in Teflon (Pine Instruments) with a geometric surface area of 0.196 cm^2. Prior to

each experiment, the two-compartment electrochemical cell was boiled in ultrapure water for 1 hour, and the electrode was polished to mirror finish with Al_2O_3 paste (particle size 0.05 µm, Buehler) on a polishing cloth (Buehler). After polishing, the electrodes were rinsed and ultrasonicated (Iskra Sonis 4, Iskra) in ultrapure water/isopropanol mixture for 3 min. 20 µL of 1 mg mL^{-1} water based, well dispersed electrocatalyst ink was pipetted on the glassy carbon electrode completely covering it and dried under ambient conditions. After the drop had dried, 5 µL of Nafion solution (ElectroChem, 5 wt% aqueous solution) diluted in isopropanol (1:50) was added. The electrode was then mounted on the rotator (Pine Instruments).

For catalysts not chemically activated, an electrochemical activation protocol was used before proceeding to the activity measurements. The working electrode was rotated at 600 rpm in Ar saturated electrolyte, 200 cycles in the potential window 0.05-1.2 V_{RHE} were conducted at a scan rate of 300 mV s^{-1}. After the activation step, the electrolyte in the cell was exchanged for the fresh one before proceeding with the next step.

For all electrocatalysts, the electrodes with electrocatalyst films were placed in the oxygen saturated electrolyte without any potential control (at the OCP) and ORR polarization curves were measured at 1600 rpm in the potential window 0.05-1.0 V_{RHE} with a scan rate of 20 mV s^{-1} immediately after measurement of ohmic resistance of the electrolyte (determined and compensated for as previously reported).[10] At the end of ORR polarization curve measurement, the electrolyte was purged with CO under potentiostatic mode (0.05 V_{RHE}) in order to ensure successful CO adsorption. Afterward the electrolyte was saturated with Ar. CO-electrooxidation was performed using the same potential window and scan rate as in ORR, but without rotation and in an Ar saturated electrolyte. After subtraction of background current due to capacitive currents, kinetic parameters were calculated at 0.95 V_{RHE} by using Koutecky-Levich equation.[11] Electrochemically active surface area ($ECSA_{CO}$) was determined by integrating the charge in CO-electrooxidation ('stripping') experiments as described in the reference.[12] All potentials are given against the reversible hydrogen electrode (RHE).

High temperature (HT) stability evaluation via accelerated degradation test (ADT) using HT Disc Electrode (HT-DE) setup

The accelerated degradation tests (ADTs) were performed in a two-compartment cell in 0.1 M $HClO_4$ (Merck, Suprapur, 70 wt%, diluted by ultrapure water (18.2 MΩ cm)) electrolyte with a conventional three-electrode system controlled by a potentiostat CompactStat (Ivium

Technologies). A hydrogen electrode (Gaskatel) was used as a reference electrode (RE), and a graphite rod (Sigma Aldrich) was used as a counter electrode (CE), The preparation of the setup and the thin films, was the same as described above. The in-house setup used is presented in **Figure S16b**.

The electrochemical potential cycling activation and ORR polarization curve and CO-electrooxidation measurements took place within the standard TF-RDE measurement setup at RT (in accordance with the same procedures described in the previous chapter). After electrochemical evaluation prior to any ADTs, the disc electrode was transferred to the HT-DE setup where 5000 cycles (0.4–1.2 V_{RHE}) with scan rate of 1 Vs^{-1} at 60 °C were performed. After the ADT, the disc electrode was transferred back to the standard TF-RDE setup and ORR polarization curve as well as CO electrooxidation were measured once again (also at RT). Stability was evaluated based on the CO-stripping performance before and after ADT.

Electrochemical evaluation with Gas Diffusion Electrode (GDE)

Electrode manufacturing – GDE manufacturing was done following the protocol used in previous work.[13] The ink for the GDE fabrication comprised of a total 1 wt% solids in a solvent mixture of 20 wt% isopropanol (IPA) in H_2O. The solid fraction was composed of 30 wt% ionomer (Nafion D520; DuPont) and of 70 wt% dealloyed electrocatalyst resulting in a gravimetric ionomer/carbon ratio of about 0.7. The ink was homogenized at 0 °C with an ultrasonic horn (Hielscher) at 60 W for 20 min. GDEs were fabricated by applying the catalyst ink onto a Freudenberg H23C8 gas diffusion media (230 µm thick) with an ultrasonic spray-coater (Biofluidix) on a heated stage at 85°C. The ink flowrate and the movement-speed of the spray-head were controlled to a deposition rate of approximately 6 μg_{Pt} cm^{-2} per deposition cycle. The Pt loading of the GDEs was measured by weighing (Sartorius Cubis, ±0.001 mg) the samples before and after the catalyst ink spray deposition.

Electrochemical half-cell and instruments – An electrochemical half-cell was specially designed to conduct measurements on GDEs as described in detail in reference.[13] For all electrochemical half-cell measurements, a VSP-300 (BioLogic) potentiostat mounted with two 2A booster boards was used.

Electrochemical measurements protocol – Before experiments with new catalysts or electrolyte, the half-cell was cleaned by boiling in 1 wt% HNO_3 solution (65 wt% EMSURE®, Merck) for 1 h. Afterward, it was boiled five times in ultrapure water (18.2 MΩ cm). Before each experiment,

the cell was boiled two times in ultrapure water again. Between the experiments the cell was always stored in ultrapure water to avoid contamination. For the half-cell measurements 1 M $HClO_4$ (70 wt% Suprapur, Merck) was used as an electrolyte. The electrochemical active surface area (ECSA) was determined by integrating the CO stripping charge at a scan rate of 200 mV s^{-1}. For ORR activity evaluation, galvanostatic steps were conducted both forward (from −0.1 mA to −4.0 A) and backward (from −4.0 A to −0.1 mA) consecutively. Thereby impedance was measured at each current step as previously reported.[13,14] All experiments were conducted under ambient conditions (101 kPa, 20 °C) and repeated three times.

Results and discussion

1. Catalyst synthesis and characterization

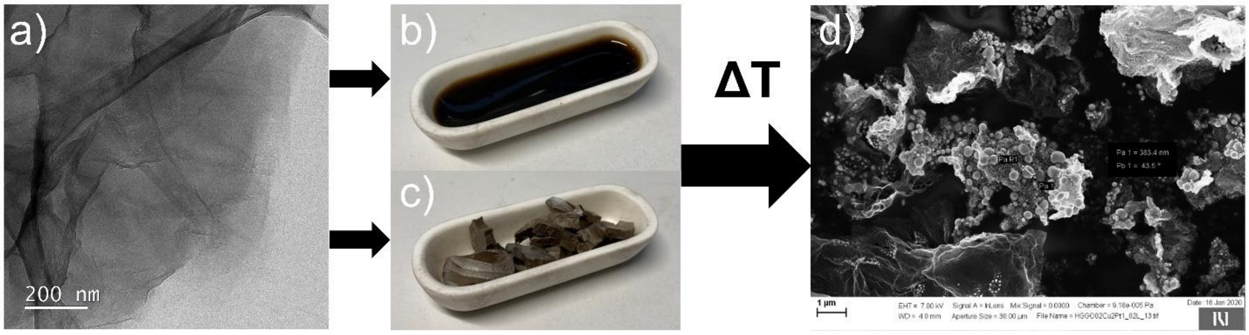

Figure S1. Comparison of two different batch approaches to the synthesis of rGO supported catalysts **(a)** TEM image of the GO **(b)** GO suspension impregnated with metal-salt precursor **(c)** freeze-dried GO suspension with impregnated metal-salt precursor **(d)** result of thermal decomposition of samples in figures **(c)** and **(b).**

Figure S1 shows a pulse combustion reactor synthesis reproduced in a batch mode inside of tube furnace. GO suspension with added adequate amount of metal acetate (M+GO/GONR **Figure S1a)** is first put in i) ceramic crucible as is-in liquid form (**Figure S1b**) or ii) freeze dried to avoid the (1.) synthesis step that involves solvent evaporation (**Figure S1c**). The crucible is put into a quartz glass tube in Ar atmosphere to avoid an oxidative environment. The crucible with the glass tube is then inserted in the tube furnace to start the synthesis. However, slower reaction conditions of a batch system involving a slower heating ramp of 10 K min^{-1} prevent the fast reaction rate that is possible to achieve in the PC reactor. Therefore, this synthesis step is prolonged to a much longer time measured in hours instead of seconds as with PC reactor. Also, the conditions in the crucible are far less ideal in comparison to PC reactor leading to a more inhomogeneous product presented in the SEM image (**Figure S1d).** Even from SEM image, it can be observed, that the particle size distribution is much broader, containing several size fractions of different nanoparticles, with the largest measuring almost 400 nm in diameter, which is too large for application in electrocatalysis.[15]

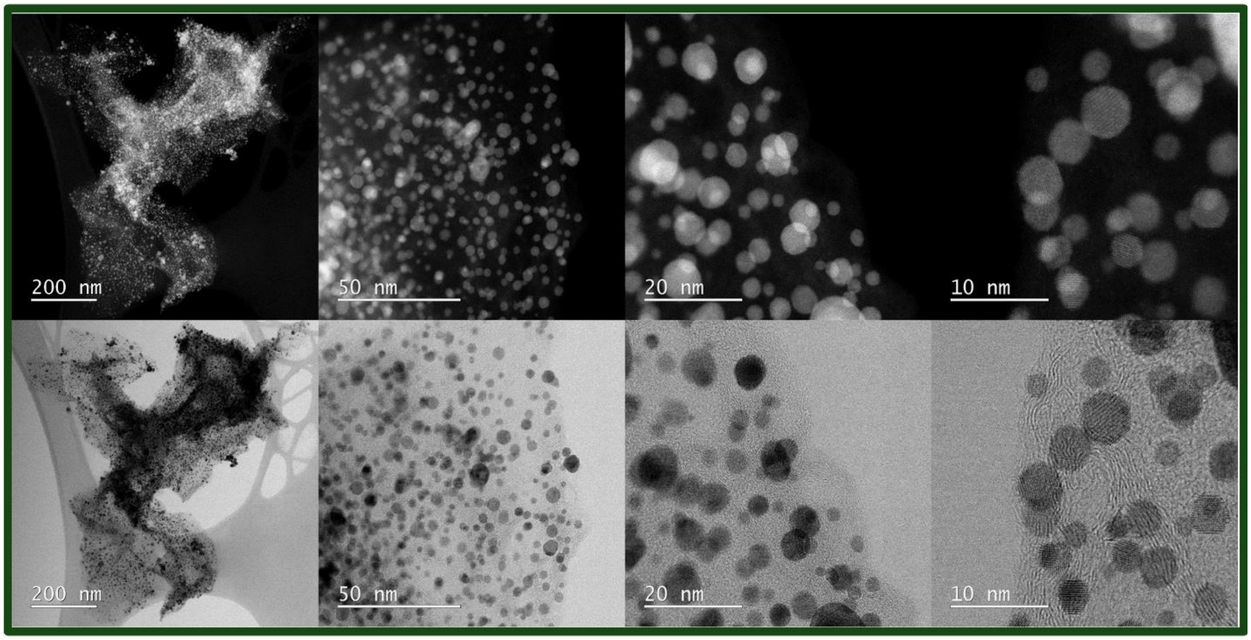

Figure S2. ADF and BF STEM imaging of PtCu/rGO electrocatalyst.

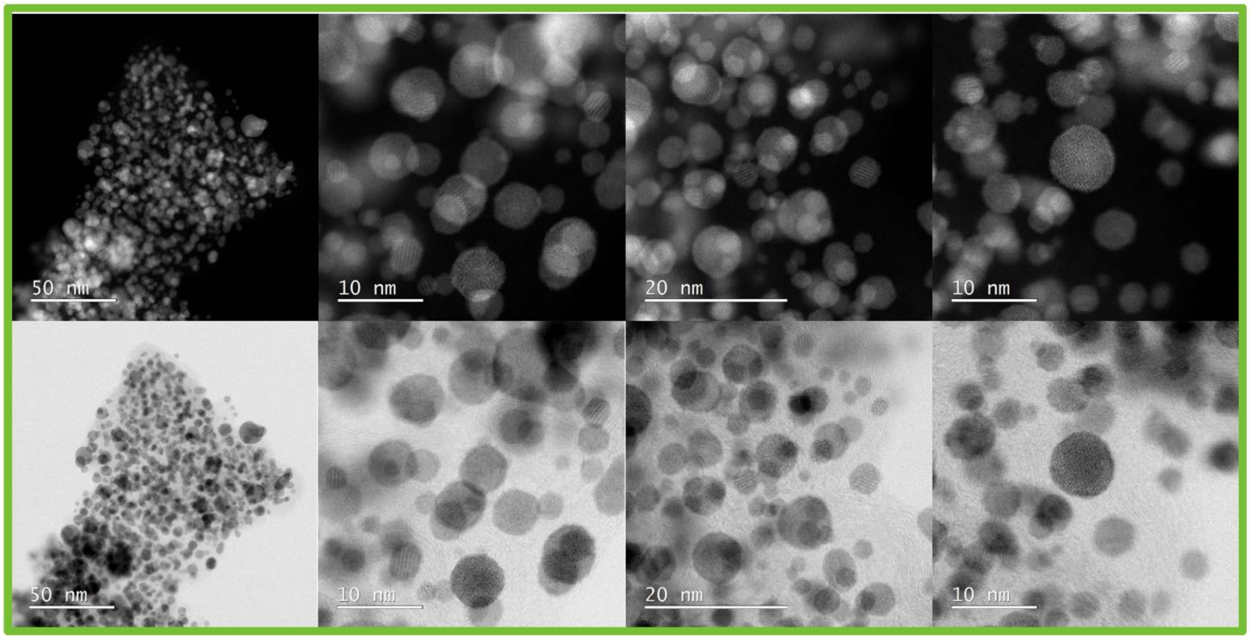

Figure S3. ADF and BF STEM imaging of PtCu/CB electrocatalyst.

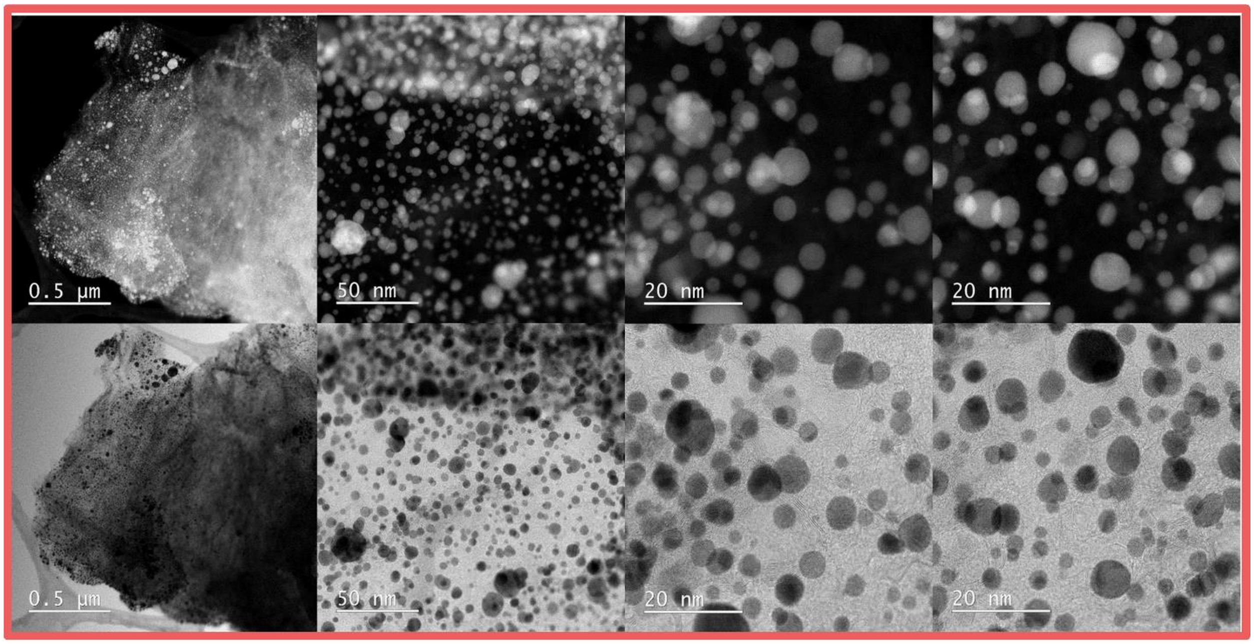

Figure S4. ADF and BF STEM imaging of PtCo/rGO electrocatalyst.

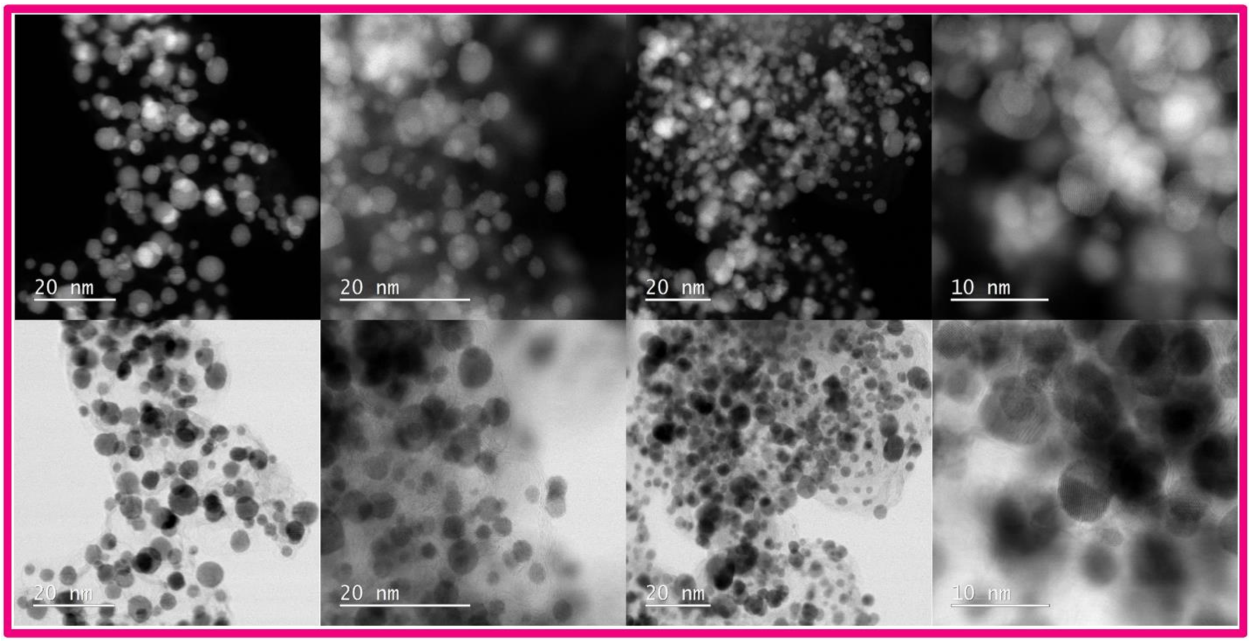

Figure S5. ADF and BF STEM imaging of PtCo/CB electrocatalyst.

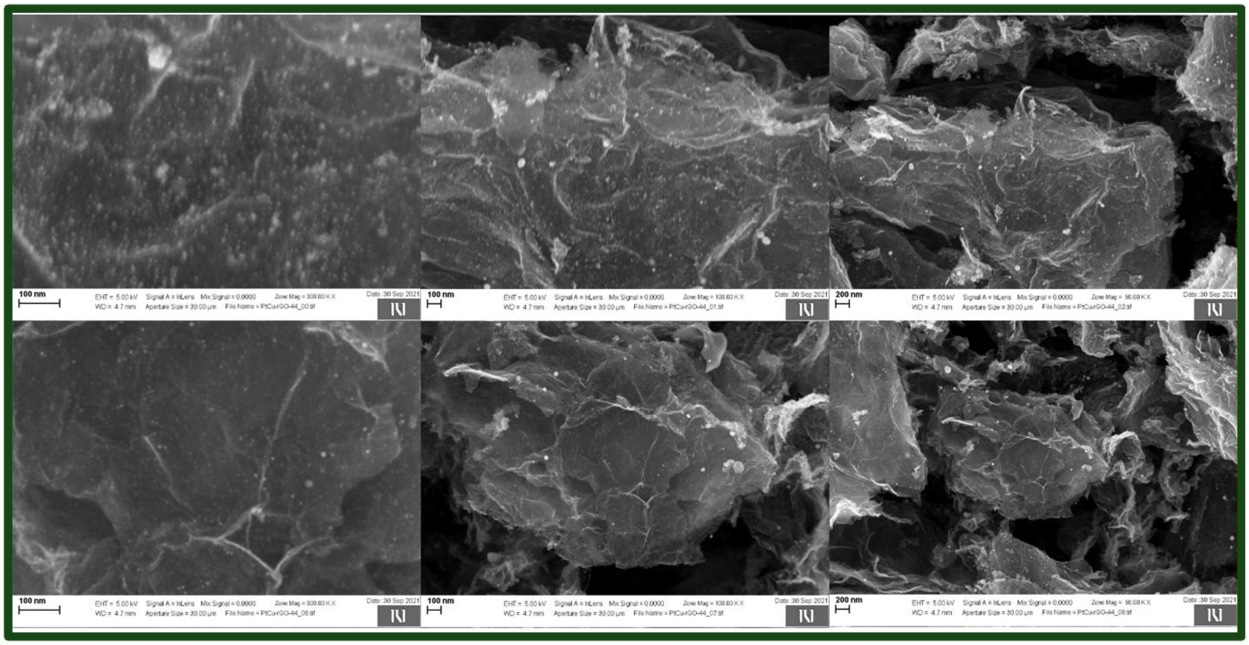

Figure S6. SEM imaging of PtCu/rGO electrocatalyst.

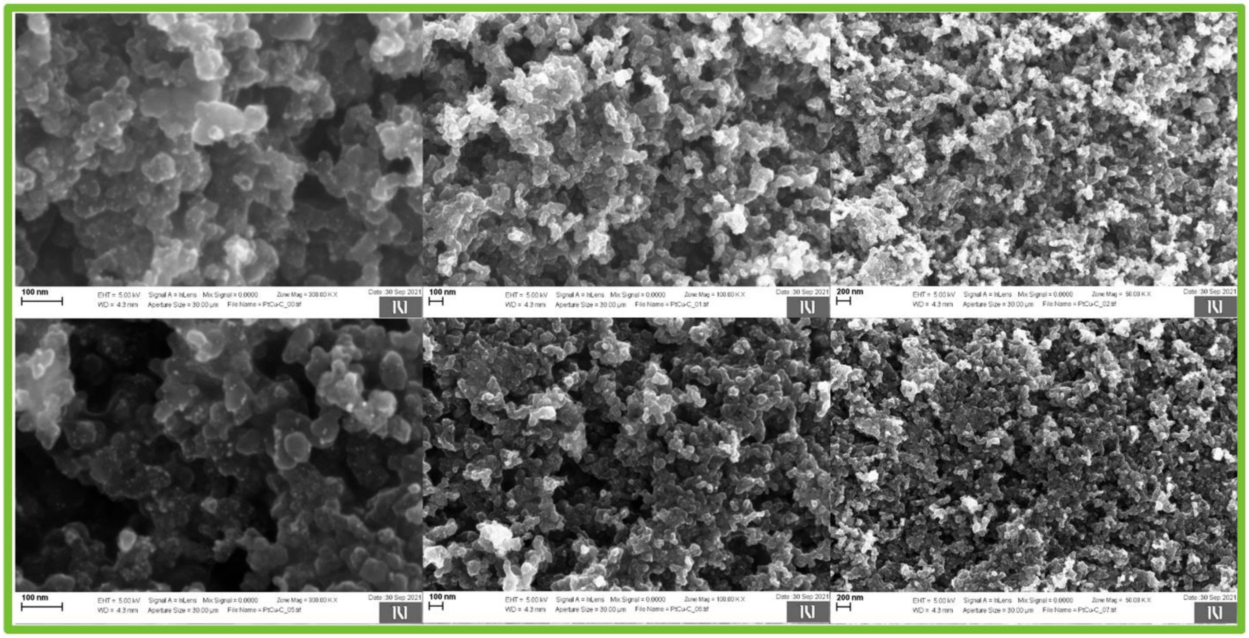

Figure S7. SEM imaging of PtCu/CB electrocatalyst.

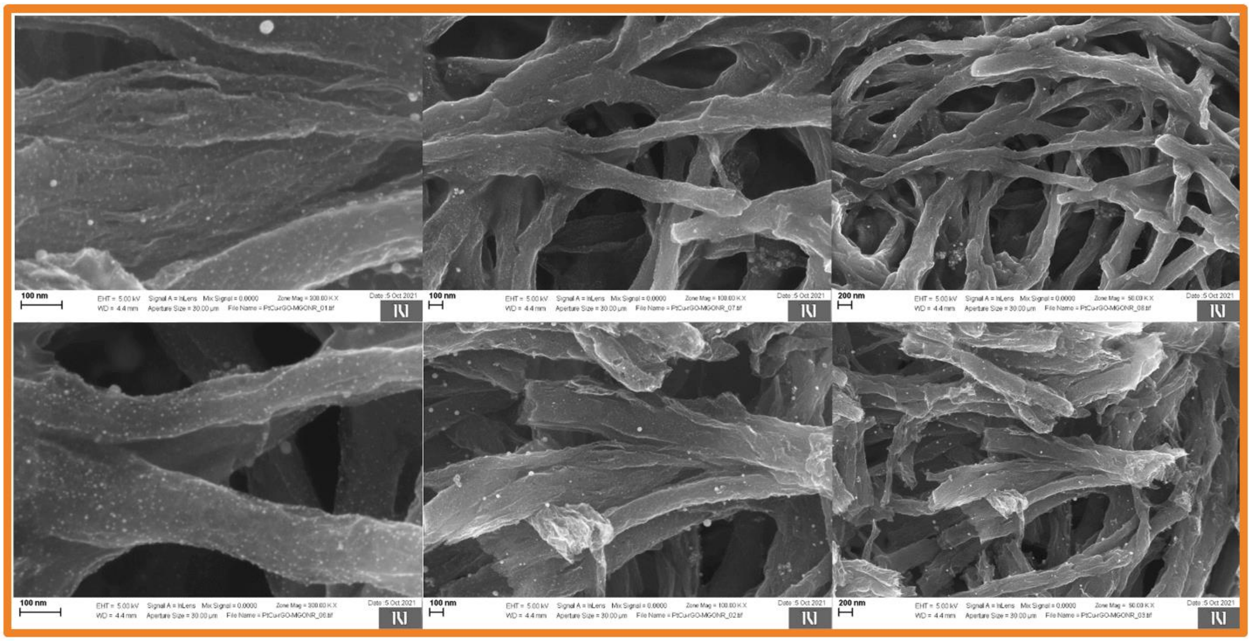

Figure S8. SEM imaging of PtCu/rGONR electrocatalyst.

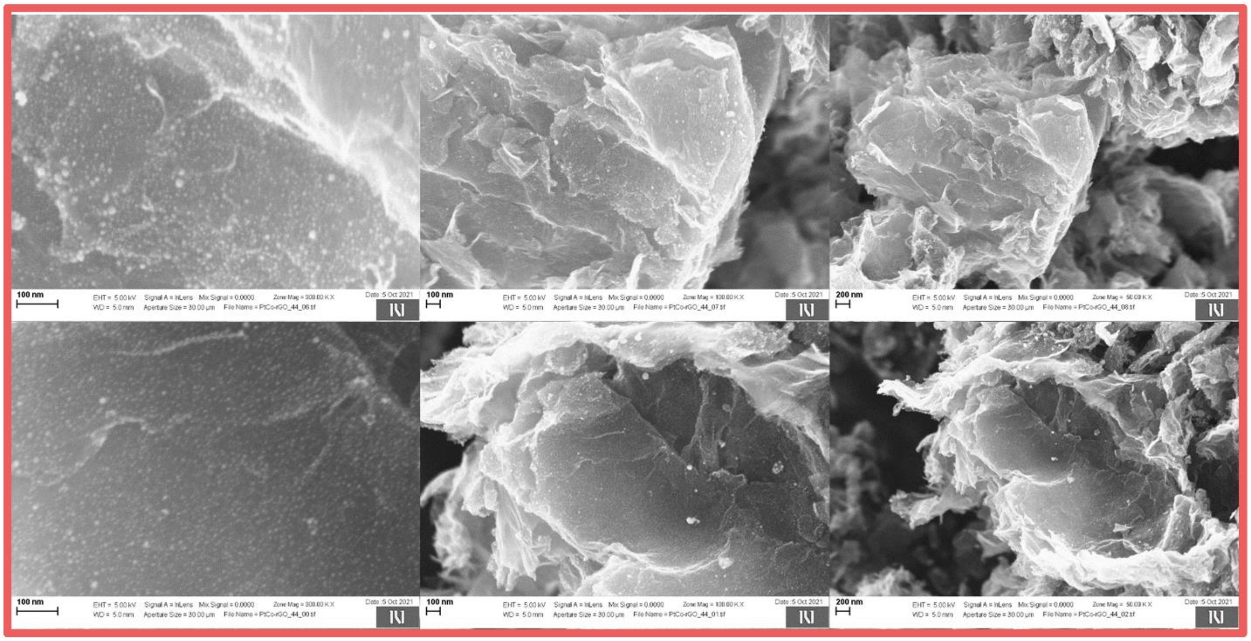

Figure S9. SEM imaging of PtCo/rGO electrocatalyst.

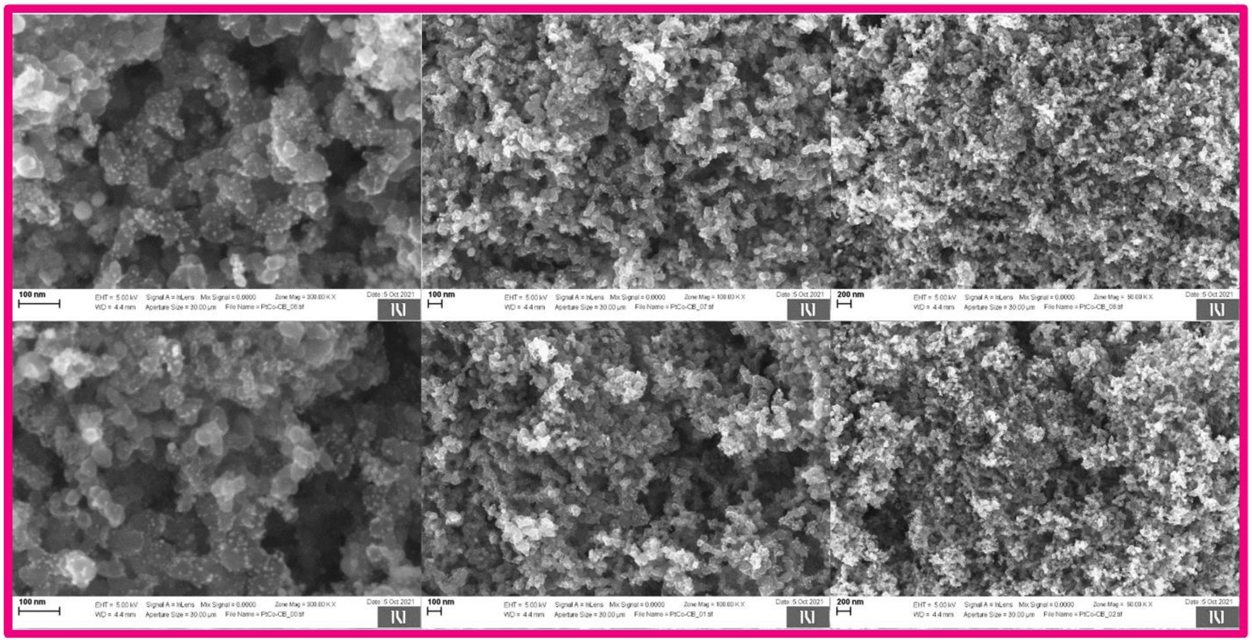

Figure S10. SEM imaging of PtCo/CB electrocatalyst.

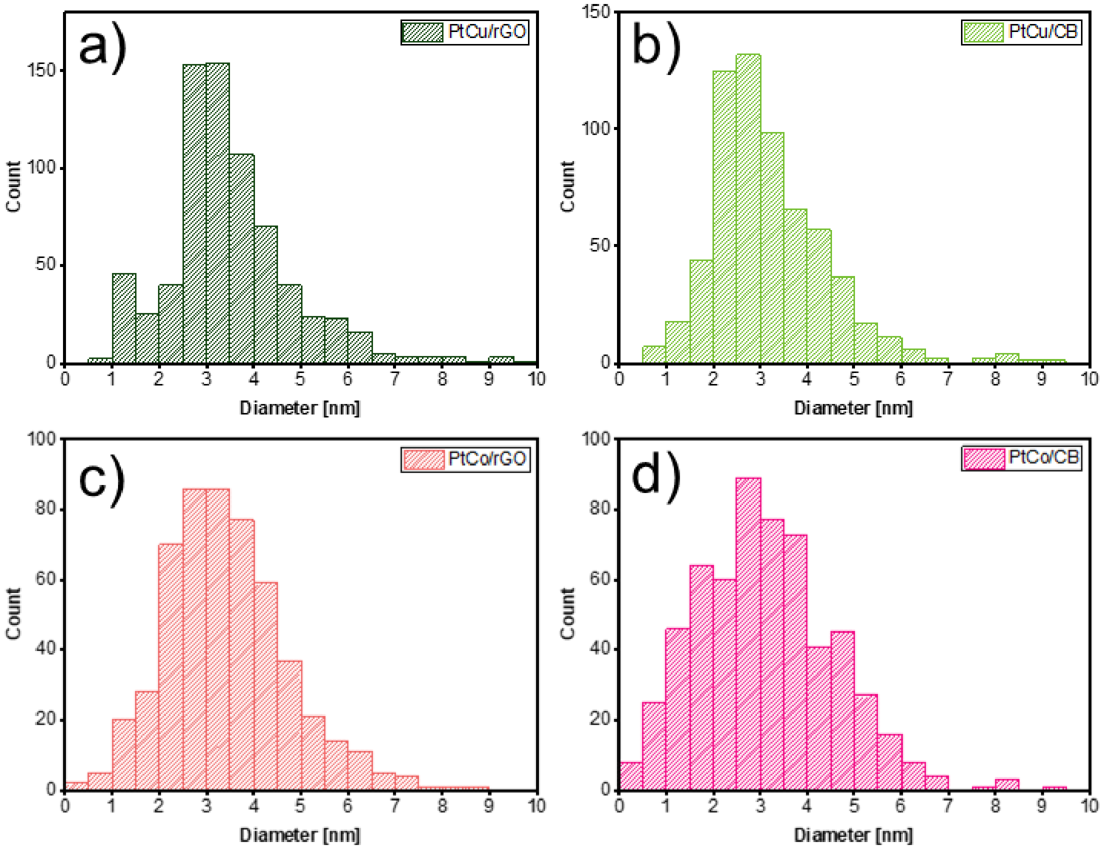

Figure S11. Particle size distribution (N > 600 particles per sample) derived from TEM images for samples: **(a)** PtCu/rGO, **(b)** PtCu/CB, **(c)** PtCo/rGO, **(d)** PtCo/CB.

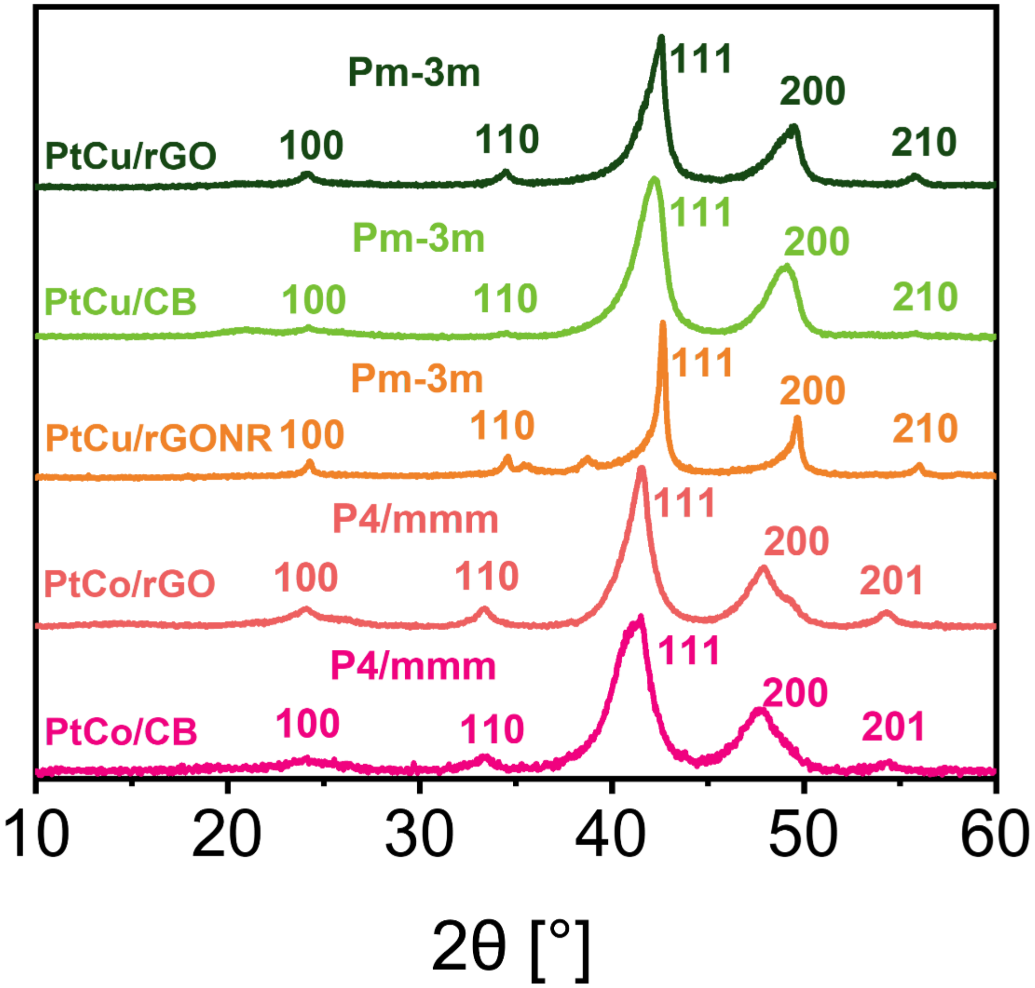

Figure S12. XRD pattern comparison of the samples synthesized within this study using the methodology presented in the experimental section.

The comparison of the X-ray diffraction (XRD) patterns shows that all PtCu analogues exhibit the presence of the cubic (Pm-3m) PtM_3 intermetallic phase, whereas PtCo analogues exhibit the tetragonal PtM intermetallic phase (P4-mmm). It can be observed that regardless of the type of carbon support used, the diffraction peaks corresponding to the metallic phases of these analogues are located at almost identical 2θ positions as well as exhibit a near identical width of the most intense peaks with a slight exception being with the PtCu/rGONR sample, where the XRD pattern is narrower in comparison to other samples, indicating slightly wider particle size distribution or presence of some larger crystallites. This specific sample (PtCu/rGONR) was synthesized without any synthesis parameter optimisation and was done the same way as other samples. The different morphology of the rGONR (**Figure S8**) could have a role in the presence of XRD pattern differences, this phenomenon should be further addressed in the upcoming studies.

Table S1. Weight percentages of metal content in the samples derived from EDX analysis.

Sample	Cu [wt%]	Co [wt%]	Pt [wt%]
PtCu/rGO	27.0	0	33.8
PtCu/CB	20.5	0	27.1
PtCu/rGONR	26.4	0	30.5
PtCo/CB	0	12.5	43.9
PtCo/rGO	0	16.8	38.5

Table S2. Weight percentages of metal content in the samples derived from ICP-OES analysis.

Sample	Cu [wt%]	Co [wt%]	Pt [wt%]
PtCu/rGO	6.36	0	40.0
PtCu/CB	5.06	0	34.45
PtCo/rGO	0	5.43	36.8
PtCo/CB	0	4.18	37.8

Table S1 shows the weight percentages of metal content derived from the EDX analysis. Here, the synthesized samples were characterized prior to the ex-situ chemical activation (de-alloying) of the samples described in the experimental section. This explains the high content of M, since the majority is removed during the activation, where also platinum rich over layer is formed. In **Table S2** presents the weight percentages of metal content analyzed using ICP-OES. Here the chemically activated (de-alloyed) samples were characterized, which explains the lower values of M. These were also the values that were used in the recipe for catalyst ink preparation [13] and for calculations of the ECSA and MA.

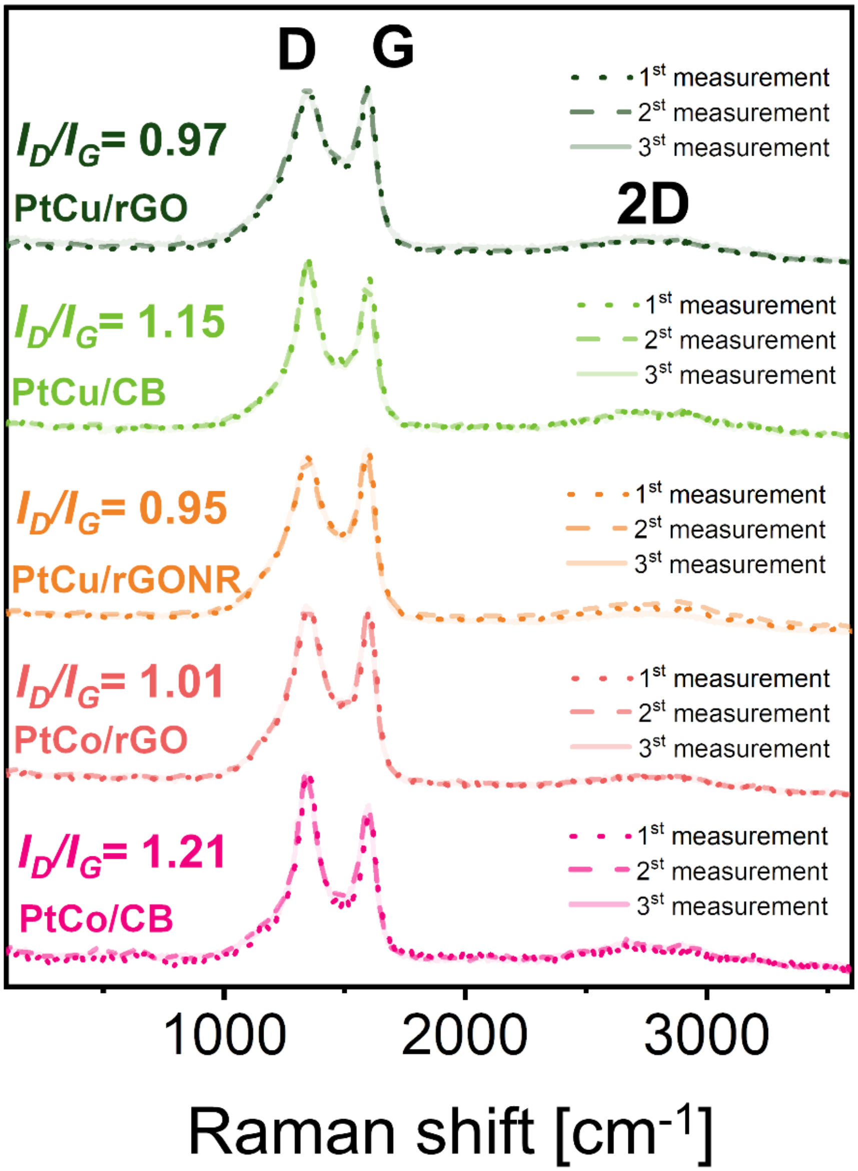

Figure S13. Raman spectra comparison showing three measurements at a different location for each sample.

Figure S13 shows Raman spectra of all the samples synthesized within this study using the methodology presented in the experimental section. Three measurements at the different location were performed for each sample to check for reproducibility. Almost identical spectra were obtained for all the samples, showing great homogeneity throughout each sample. Moreover, it can be observed that the samples where GDs are utilized as support show noticeably lower I_D/I_G ratio than the CB containing samples. This is agrees well with previous reports, as GDs are expected to contain fewer structural defects and a higher degree of graphitisation than partially graphitised CBs.[16]

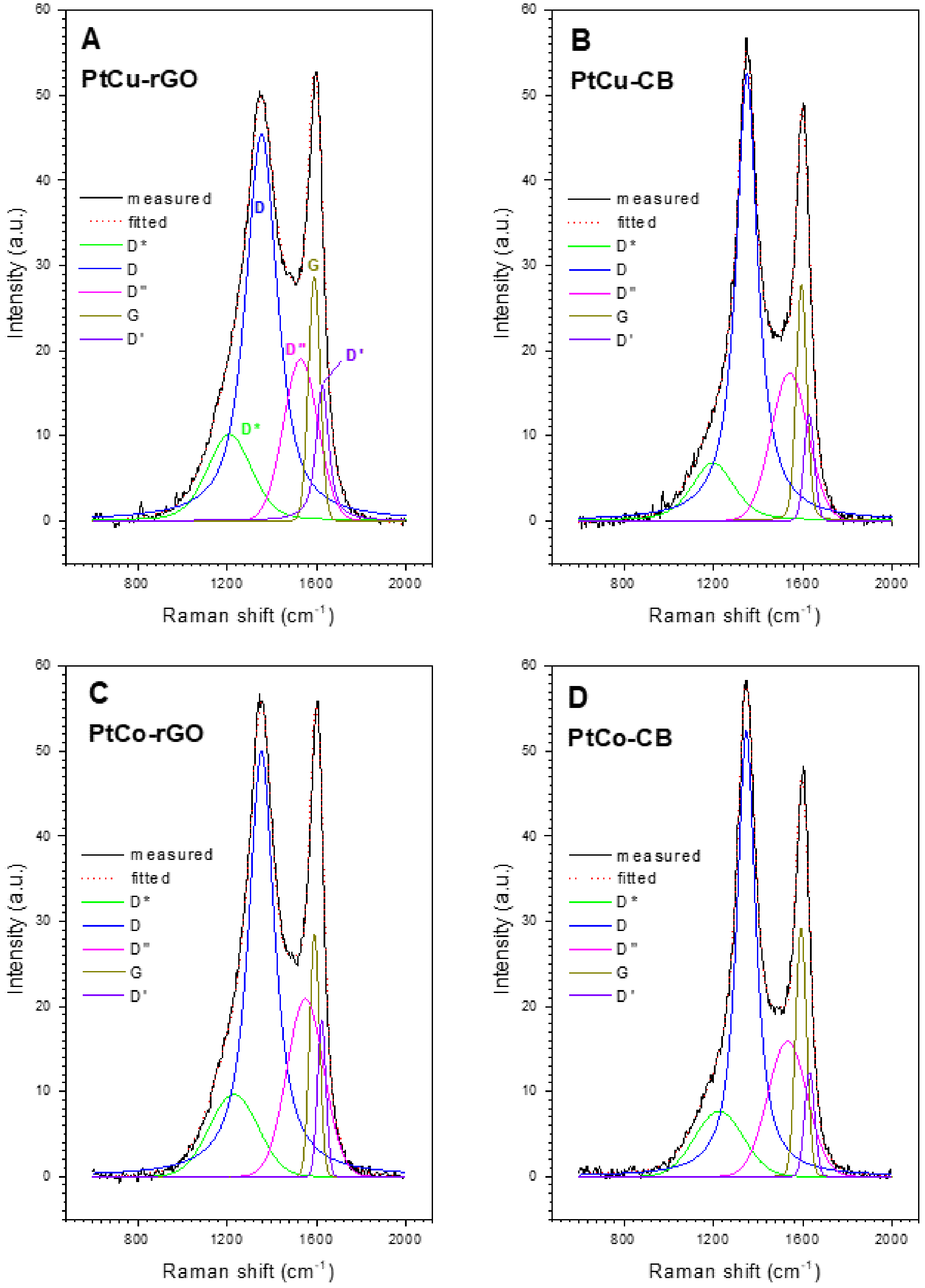

Figure S14. Deconvoluted Raman spectra of: A) PtCu/rGO, B) PtCu/CB, C) PtCo/rGO and D) PtCu/CB.

The Raman spectra of samples in the spectral range 600 – 2000 cm^{-1} were deconvoluted using GRAMS/AI, version 9.3 program (Thermo Fisher Scientific Inc.). The spectra were fitted with five bands which have already been described in the literature.[17–21] The Gaussian function was applied for fitting of D'' band while mixed Gaussian-Lorentzian function was used for fitting of other four bands (i.e., D*, D, G and D'). The fitting procedure enabled a more profound insight into the sample structure and confirmed the I_D/I_G ratio trend from the **Figure 2f**. Namely, the I_D/I_G ratio of fitted D and G bands remained higher for samples PtCu/rGO, PtCo/rGO in comparison to samples PtCu/CB, PtCo/CB. Our future work will though be oriented towards detailed analysis of deconvoluted peaks, amorphization trajectories and estimation of defects.

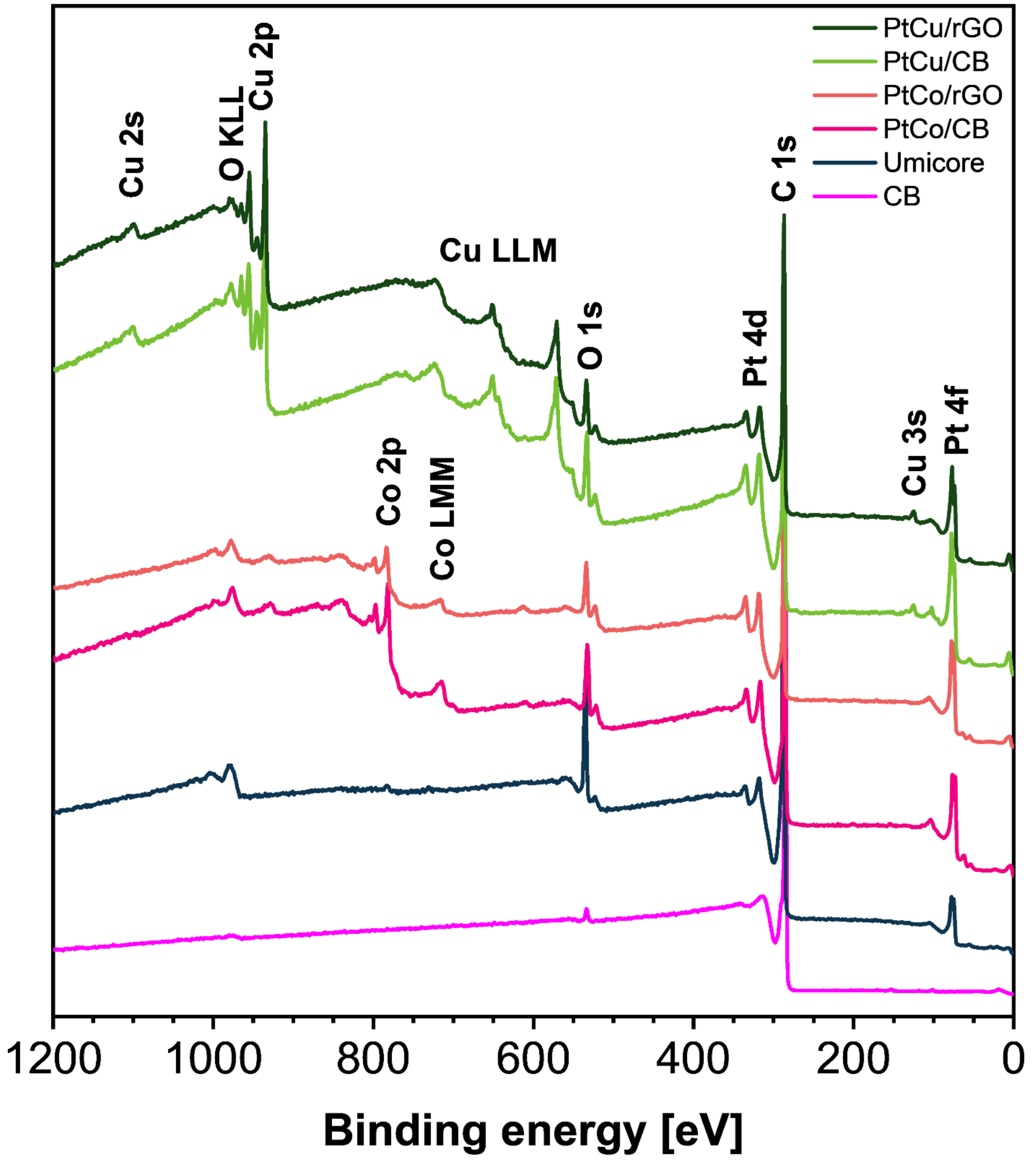

Figure S15. XPS survey spectra of the powder samples.

Figure S15 shows the XPS survey spectra of the synthesized samples presented in this study, Elyst Pt30 0690 (Umicore), and an additional sample of as received carbon black, CB (Ketjen Black EC300J), which was used to synthesize the CB supported catalysts. The catalyst samples consist of C, O, Pt, and M (Cu in the case of PtCu analogues and Co in the case of PtCo analogues and

Umicore). CB sample contains only C and O. The difference in shape of survey can be observed when PtCo analogues are compared with Umicore, where the peaks for metal phase are less pronounced. This may be since the as-received Umicore sample is already in active form (dealloyed), compared to the synthetically produced PtCo analogues (non-dealloyed), which still have a higher metal content. This also agrees well with the EDX analysis in **Table S1** and the ICP-OES data in **Table S2**.

Figure S16 shows the procedure for processing the Auger C KLL spectra. The noise in the obtained Auger C KLL spectra (**Figure S16a**) can lead to partial uncertainty in the determination of the D-parameter. This problem was successfully solved by smoothing the signal before differentiation in the next step. CasaXPS (Casa Software Ltd, Teignmouth, United Kingdom) software was used for this purpose. The Savitzky-Golay (SG) smoothing function with 2nd order polynomial (quadratic; p=2) with selection width (w=9) was applied, resulting in smoothed signals (**Figure S16b**). 1st-order differentiation was applied using quadratic SG function with a selection width (w=5) to obtain the 1st derivative of the Auger C KLL spectra (**Figure S16c**). Similar data processing was applied to the data in **Figures S16d-e** with a difference in the parameters of the smoothing function. Here, a 4th polynomial order smoothing function (quartic; p=4) SG with a selection width (w=15) was used to obtain a smoothed signal shown in **Figure S16d**. The 1st order derivative was applied using SG quadratic function with a selection width (w=5) to obtain the 1st 1st derivative of the Auger C KLL spectra (**Figure S16e**). To obtain the value of the D parameter (see **Figures S16c, e**), the eV value at the maxima of the 1st derivative was subtracted from the x value at the minima for each spectrum. The sp^2 content was calculated using the linear relationship between the D-parameter and sp^2 content. To determine the linear function linking the D-parameter to sp^2 content, the value of the D-parameter for 100.0% sp^2 material was 23.0, and for 100.0% sp^3 material was 14.2.[22,23] The determined sp^2 content in the samples is shown in **Table S3**. The sp^2 content of 76.6%, 77.3% for GD supported catalysts is noticeably higher than for CB analogues (70.0%, 67.5%) in case of smoothing using the quartic SG function. Moreover, the sp^2 content values are very consistent within each material group. Moreover, the sp^2 content of bare carbon black (sample CB) agrees well with the final catalysts where CB was used as the carbon support. This is also an indication that the CB material did not chemically change during the synthesis process. To further check the sp^2 content in each sample, deconvolution of the high-resolution C 1s spectra was performed (**Figure S17**). A Shirley background was used in all cases, with the

maximum of the C 1s spectra fixed at a binding energy of 284.5 eV (due to higher sp^2 content than sp^3). The full width at half maxima (FWHM) of all deconvoluted peaks was kept the same for fitting. In the fitting procedure, the ratio of sp^2 and sp^3 content as determined by the D-parameter method was fixed for all samples (**Table S3**). The fitted curve agrees well with the measured data, indicating the adequacy of the procedure used.

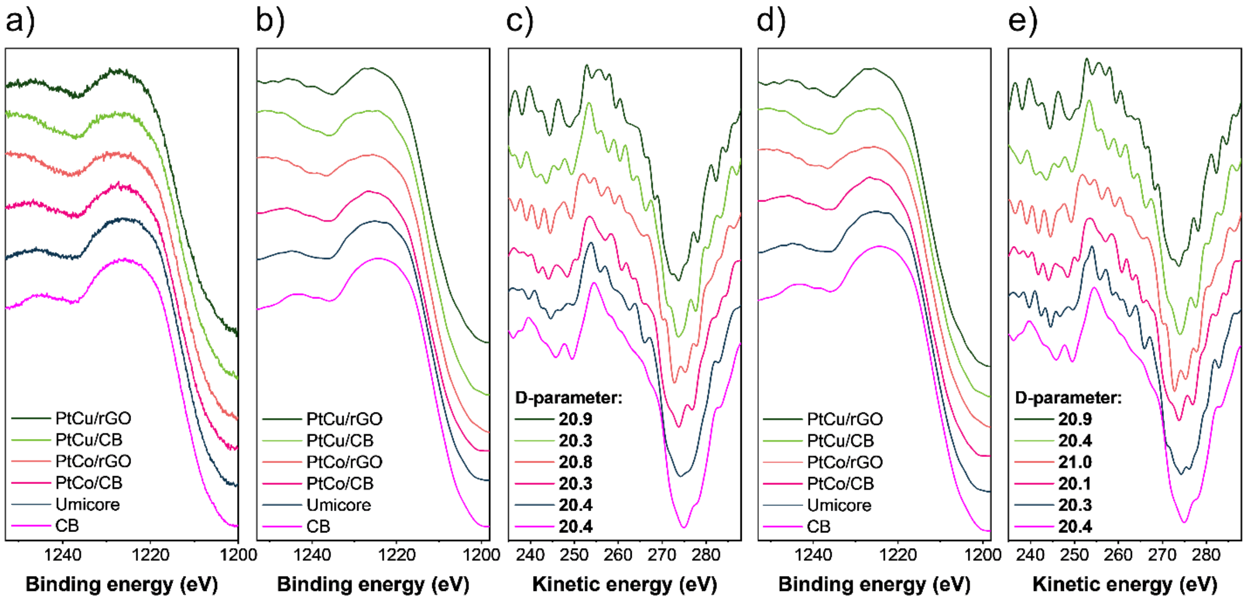

Figure S16. The Auger C KLL spectra processing; **(a)** measured Auger C KLL spectra, **(b)** smoothed Auger C KLL spectra using SG quadratic (9) function, **(c)** 1st derivative of C KLL Auger spectra using SG quadratic (5) function, **(d)** smoothed Auger C KLL spectra using SG quartic (15) function, **(e)** 1st derivative of Auger C KLL Auger spectra using SG quadratic (5) function.

Table S3. Calculated sp^2 in % values obtained with two different smoothing modes with different w/p ratios.

**sample**	***sp*^{2} [%]**	***sp*^{2} [%]**
**PtCu/rGO**	76.2	76.6
**PtCu/CB**	69.7	70.0
**PtCo/rGO**	74.5	77.3
**PtCo/CB**	69.2	67.5
**Umicore**	70.2	69.2
**CB**	70.5	70.6
**smoothing:**	SG quadratic (9)	SG quartic (15)
**differentiation:**	SG quadratic (5)	SG quadratic (5)
**w/p ratio:**	**4.5**	**3.75**

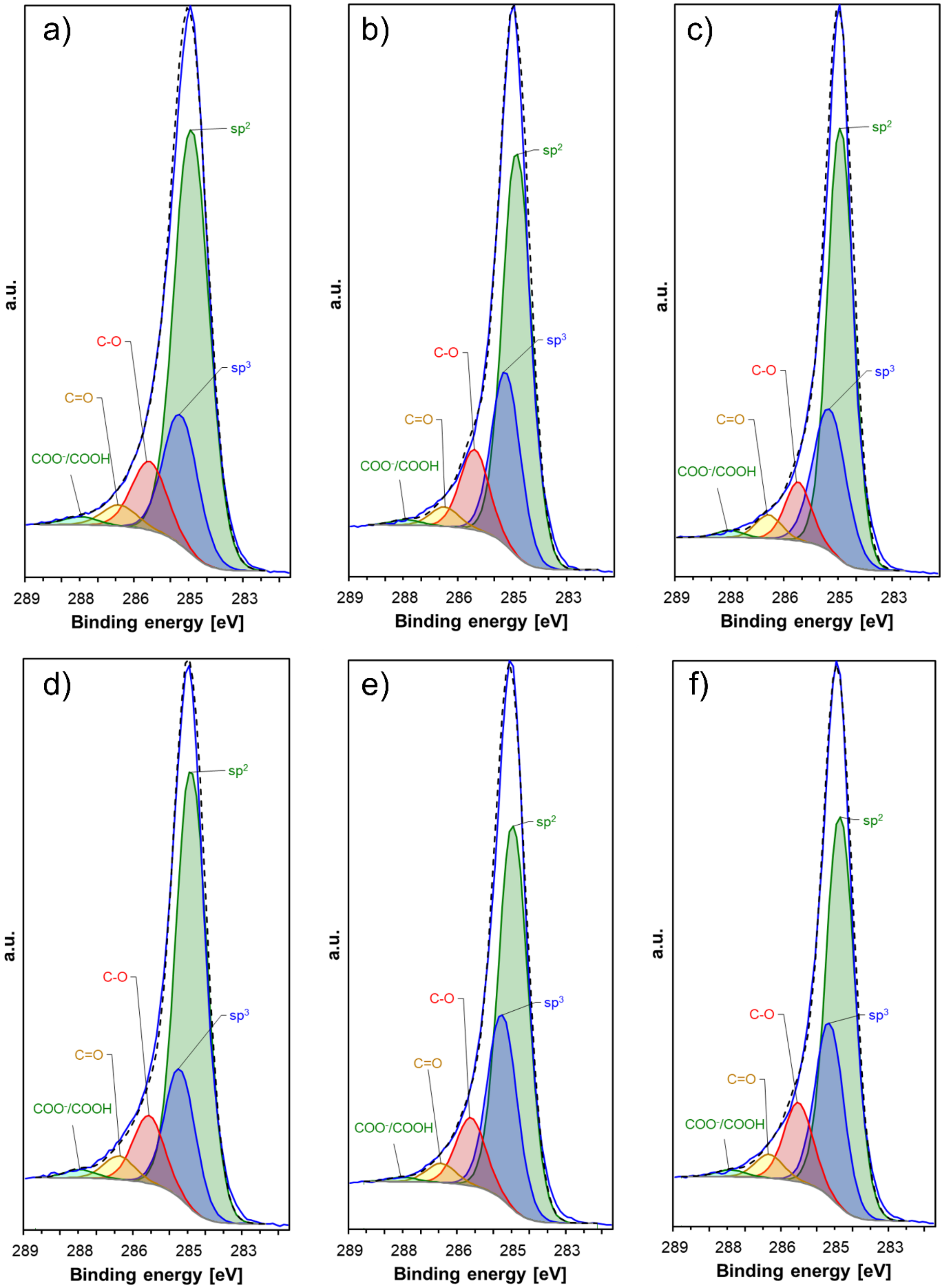

Figure S17. Deconvolution of C 1s HR spectra for the samples: **(a)** PtCu/rGO, **(b)** PtCu/CB, **(c)** CB, **(d)** PtCo/rGO, **(e)** PtCo/CB, and **(f)** Umicore.

2. Rotating disc electrode results

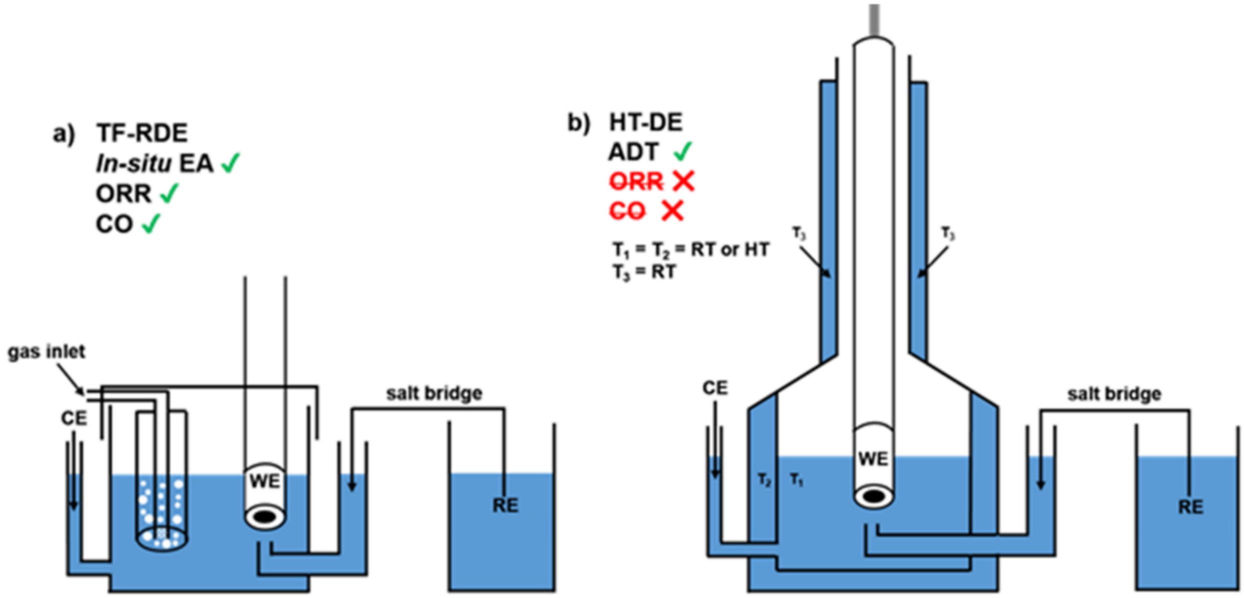

Figure S18. Comparison of **(a)** conventional, two-compartment TF-RDE setup and **(b)** in-house designed HT-DE setup for performing ADTs at elevated temperatures using a thermostat.[24]

Figure S18a presents the scheme of a conventional, two compartment TF-RDE setup which was used for $ECSA_{CO}$ and MA determination. **Figure S18b** presents a scheme of an in-house designed HT-DE setup which was used to perform ADTs. In this case the electrolyte evaporation is mitigated via a refluxing column. The electrocatalyst evaluation such as electrochemical activation (in situ EA) as well as ORR polarization curve or CO-electrooxidation/HUPD CV measurements take place in the TF-RDE setup at RT, while disc electrode is transferred to the HT-DE setup for ADT at elevated temperatures (e.g., 60 °C). Performance after successful ADT is re-evaluated in the normal two compartment TF-RDE setup (**Figure S18a**). $ECSA_{CO}$ values after EA (before ADT) is shown in **Figure S19a**, whereas $ECSA_{CO}$ values after ADT are presented in **Figure S19b**. Examples of CO-stripping measurements from which $ECSA_{CO}$ data was derived from are presented in **Figure S20.**

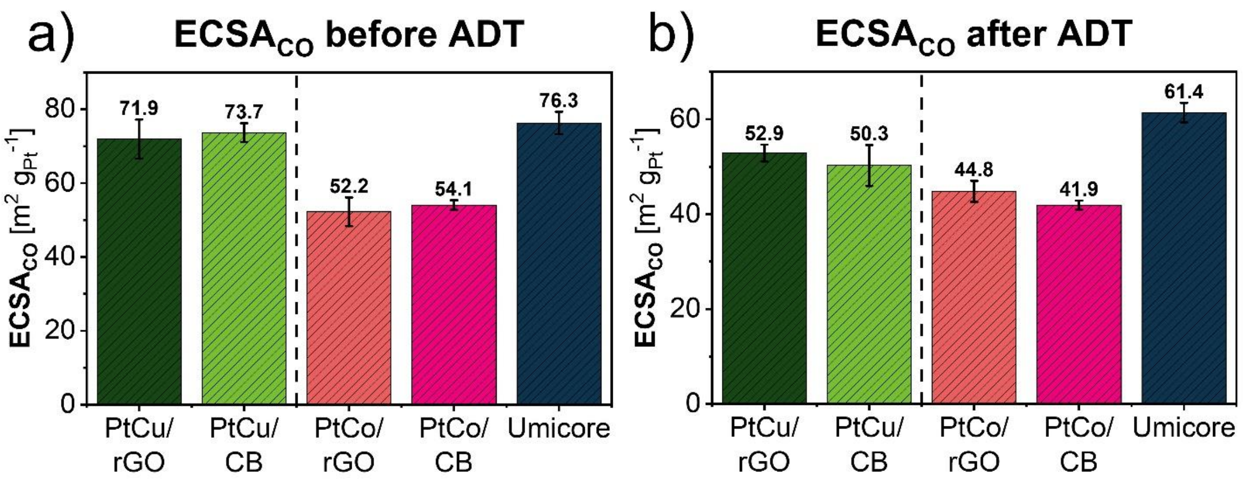

Figure S19. **(a)** ECSA$_{CO}$ values after electrochemical activation (EA) **(b)** ECSA$_{CO}$ values after accelerated degradation test (ADT).

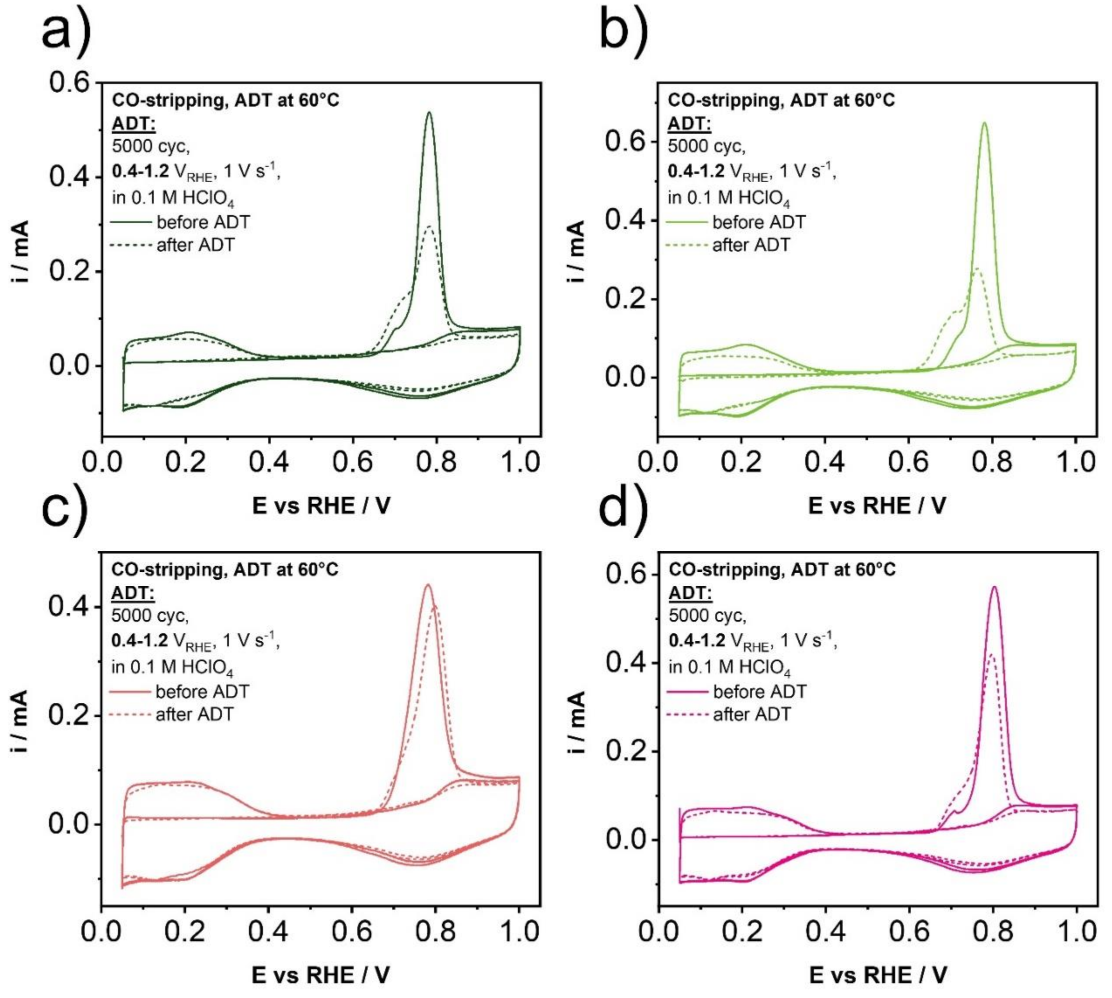

Figure S20. CO-stripping before and after ADT for samples: **(a)** PtCu/rGO, **(b)** PtCu/CB, **(c)** PtCo/rGO, **(d)** PtCo/CB.

Figure S21a is presenting MA values at 0.95 V_{RHE} after EA (before ADT), whereas MA values after ADT are presented in **Figure S21b**. For all the samples three measurements were conducted which included the measurement process in the following order: in-situ EA, ORR, CO-electrooxidation, ADT, ORR, CO-electrooxidation. Error bars in **Figure S19** and **figure S21** are showing standard deviation calculated based on all three measurements to show for reproducibility of the results.

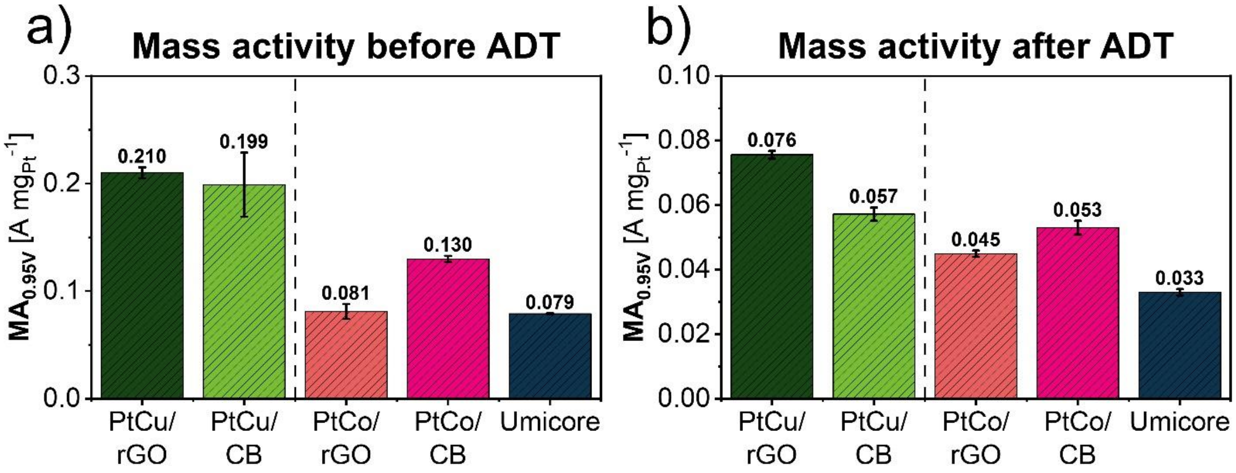

Figure S21. **(a)** Mass activity (MA) at 0.95 V_{RHE} after electrochemical activation (EA) **(b)** Mass activity at 0.95 V_{RHE} after accelerated degradation test (ADT).

References

(1) Marcano, D. C.; Kosynkin, D. V.; Berlin, J. M.; Sinitskii, A.; Sun, Z.; Slesarev, A.; Alemany, L. B.; Lu, W.; Tour, J. M. Improved Synthesis of Graphene Oxide. *ACS Nano* **2010**, *4* (8), 4806–4814. https://doi.org/10.1021/nn1006368.

(2) Pavko, L.; Gatalo, M.; Križan, G.; Križan, J.; Ehelebe, K.; Ruiz-Zepeda, F.; Šala, M.; Dražić, G.; Geuß, M.; Kaiser, P.; Bele, M.; Kostelec, M.; Đukić, T.; Van de Velde, N.; Jerman, I.; Cherevko, S.; Hodnik, N.; Genorio, B.; Gaberšček, M. Toward the Continuous Production of Multigram Quantities of Highly Uniform Supported Metallic Nanoparticles and Their Application for Synthesis of Superior Intermetallic Pt-Alloy ORR Electrocatalysts. *ACS Appl. Energy Mater.* **2021**. https://doi.org/10.1021/acsaem.1c02570.

(3) Križan, G.; Križan, J.; Dominko, R.; Gaberšček, M. Pulse Combustion Reactor as a Fast and Scalable Synthetic Method for Preparation of Li-Ion Cathode Materials. *J. Power Sources* **2017**, *363*, 218–226. https://doi.org/https://doi.org/10.1016/j.jpowsour.2017.07.083.

(4) Lazarević, Z. Ž.; Križan, G.; Križan, J.; Milutinović, A.; Ivanovski, V. N.; Mitrić, M.; Gilić, M.; Umićević, A.; Kuryliszyn-Kudelska, I.; Romčević, N. Ž. Characterization of LiFePO4 Samples Obtained by Pulse Combustion under Various Conditions of Synthesis. *J. Appl. Phys.* **2019**, *126* (8), 85109. https://doi.org/10.1063/1.5100358.

(5) Križan, G.; Križan, J.; Bajsić, I.; Gaberšček, M. Control of a Pulse Combustion Reactor with Thermoacoustic Phenomena. *Instrum. Sci. Technol.* **2018**, *46* (1), 43–57. https://doi.org/10.1080/10739149.2017.1320288.

(6) Gatalo, M.; Bele, M.; Ruiz-Zepeda, F.; Šest, E.; Šala, M.; Kamšek, A. R.; Maselj, N.; Galun, T.; Jovanovič, P.; Hodnik, N.; Gaberšček, M. A Double-Passivation Water-Based Galvanic Displacement Method for Reproducible Gram-Scale Production of High-Performance Platinum-Alloy Electrocatalysts. *Angew. Chemie* **2019**, *131* (38), 13400–13404. https://doi.org/10.1002/ange.201903568.

(7) Gatalo, M.; Ruiz-Zepeda, F.; Hodnik, N.; Dražić, G.; Bele, M.; Gaberšček, M. Insights into Thermal Annealing of Highly-Active PtCu3/C Oxygen Reduction Reaction Electrocatalyst: An in-Situ Heating Transmission Electron Microscopy Study. *Nano Energy* **2019**, *63*, 103892. https://doi.org/https://doi.org/10.1016/j.nanoen.2019.103892.

(8) Kongkanand, A.; Wagner, F. *High-Activity Dealloyed Catalysts, 2014 DOE Hydrogen and Fuel Cells Program Review, Washington, D. C., June 16–20,*; 2014.

(9) Myers, D.; Kariuki, N.; Ahluwalia, R.; Xiaohua, W.; Cetinbas, C. F.; Peng, J.-K. *Rationally Designed Catalyst Layers for PEMFC Performance Optimization, 2016 DOE Hydrogen and Fuel Cells Program Review, Washington, D. C., June 6–10, 2016.*

(10) van der Vliet, D.; Strmcnik, D. S.; Wang, C.; Stamenkovic, V. R.; Markovic, N. M.; Koper, M. T. M. On the Importance of Correcting for the Uncompensated Ohmic Resistance in Model Experiments of the Oxygen Reduction Reaction. *J. Electroanal. Chem.* **2010**, *647* (1), 29–34. https://doi.org/https://doi.org/10.1016/j.jelechem.2010.05.016.

(11) Bard, A. J.; Faulkner, L. R. *Electrochemical Methods: Fundamentals and Applications*; Wiley, 2000.

(12) Mayrhofer, K. J. J.; Strmčnik, D.; Blizanac, B. B.; Stamenković, V. R.; Arenz, M.; Marković, N. M. Measurement of Oxygen Reduction Activities via the Rotating Disc Electrode Method: From Pt Model Surfaces to Carbon-Supported High Surface Area Catalysts. *Electrochim. Acta* **2008**, *53*, 3181–3188. https://doi.org/http://dx.doi.org/10.1016/j.electacta.2007.11.057.

(13) Ehelebe, K.; Seeberger, D.; Paul, M. T. Y.; Thiele, S.; Mayrhofer, K. J. J.; Cherevko, S. Evaluating Electrocatalysts at Relevant Currents in a Half-Cell: The Impact of Pt Loading on Oxygen Reduction Reaction. *J. Electrochem. Soc.* **2019**, *166* (16), F1259–F1268. https://doi.org/10.1149/2.0911915jes.

(14) Pinaud, B. A.; Bonakdarpour, A.; Daniel, L.; Sharman, J.; Wilkinson, D. P. Key Considerations for High Current Fuel Cell Catalyst Testing in an Electrochemical Half-Cell. *J. Electrochem. Soc.* **2017**, *164* (4), F321–F327. https://doi.org/10.1149/2.0891704jes.

(15) Kongkanand, A.; Mathias, M. F. The Priority and Challenge of High-Power Performance of Low-Platinum Proton-Exchange Membrane Fuel Cells. *J. Phys. Chem. Lett.* **2016**, *7* (7), 1127–1137. https://doi.org/10.1021/acs.jpclett.6b00216.

(16) Zhang, S.; Cui, Y.; Wu, B.; Song, R.; Song, H.; Zhou, J.; Chen, X.; Liu, J.; Cao, L. Control of Graphitization Degree and Defects of Carbon Blacks through Ball-Milling. *RSC Adv.* **2014**, *4* (1), 505–509. https://doi.org/10.1039/C3RA44530E.

(17) López-Díaz, D.; López Holgado, M.; García-Fierro, J. L.; Velázquez, M. M. Evolution of the Raman Spectrum with the Chemical Composition of Graphene Oxide. *J. Phys. Chem. C* **2017**, *121* (37), 20489–20497. https://doi.org/10.1021/acs.jpcc.7b06236.

(18) Karačić, D.; Gutić, S. J.; Vasić, B.; Mirsky, V. M.; Skorodumova, N. V.; Mentus, S. V.; Pašti, I. A. Electrochemical Reduction of Thin Graphene-Oxide Films in Aqueous Solutions – Restoration of Conductivity. *Electrochim. Acta* **2022**, *410* (January), 140046. https://doi.org/10.1016/j.electacta.2022.140046.

(19) Claramunt, S.; Varea, A.; López-Díaz, D.; Velázquez, M. M.; Cornet, A.; Cirera, A. The Importance of Interbands on the Interpretation of the Raman Spectrum of Graphene Oxide. *J. Phys. Chem. C* **2015**, *119* (18), 10123–10129. https://doi.org/10.1021/acs.jpcc.5b01590.

(20) Rafailović, L. D.; Jovanović, A. Z.; Gutić, S. J.; Wehr, J.; Rentenberger, C.; Trišović, T. L.; Pašti, I. A. New Insights into the Metallization of Graphene-Supported Composite Materials-from 3D Cu-Grown Structures to Free-Standing Electrodeposited Porous Ni Foils. *ACS Omega* **2022**, *7* (5), 4352–4362. https://doi.org/10.1021/acsomega.1c06145.

(21) Sadezky, A.; Muckenhuber, H.; Grothe, H.; Niessner, R.; Pöschl, U. Raman Microspectroscopy of Soot and Related Carbonaceous Materials: Spectral Analysis and Structural Information. *Carbon N. Y.* **2005**, *43* (8), 1731–1742. https://doi.org/10.1016/j.carbon.2005.02.018.

(22) Lascovich, J. C.; Giorgi, R.; Scaglione, S. Evaluation of the Sp2/Sp3 Ratio in Amorphous Carbon Structure by XPS and XAES. *Appl. Surf. Sci.* **1991**, *47* (1), 17–21. https://doi.org/https://doi.org/10.1016/0169-4332(91)90098-5.

(23) Morgan, D. J. Comments on the XPS Analysis of Carbon Materials. *C* **2021**, *7* (3), 51. https://doi.org/10.3390/c7030051.

(24) Maselj, N.; Gatalo, M.; Ruiz-Zepeda, F.; Kregar, A.; Jovanovič, P.; Hodnik, N.; Gaberšček, M. The Importance of Temperature and Potential Window in Stability Evaluation of Supported Pt-Based Oxygen Reduction Reaction Electrocatalysts in Thin Film Rotating Disc Electrode Setup. *J. Electrochem. Soc.* **2020**. https://doi.org/10.1149/1945-7111/aba4e6.
